# Nuclear Magnetic Resonance Metabolomics Reveals Qualitative and Quantitative Differences in the Composition of Human Breast Milk and Milk Formulas

**DOI:** 10.3390/nu12040921

**Published:** 2020-03-27

**Authors:** Dorota Garwolińska, Weronika Hewelt-Belka, Agata Kot-Wasik, Ulrik Kræmer Sundekilde

**Affiliations:** 1 Department of Analytical Chemistry, Faculty of Chemistry, Gdańsk University of Technology, Gabriela Narutowicza 11/12, 80-233 Gdańsk, Polandagawasik@pg.edu.pl (A.K.-W.); 2 Department of Food Science, Faculty of Technical Sciences, Aarhus University, Agro Food Park 48, DK-8200 Aarhus N, Denmark; uksundekilde@food.au.dk

**Keywords:** human breast milk, human milk composition, human milk metabolites, breastfeeding, infant feeding, infant formula, NMR metabolomics, foodomics

## Abstract

Commercial formula milk (FM) constitutes the best alternative to fulfill the nutritional requirements of infants when breastfeeding is precluded. Here, we present the comparative study of polar metabolite composition of human breast milk (HBM) and seven different brands of FM by nuclear magnetic resonance spectroscopy. The results of the multivariate data analysis exposed qualitative and quantitative differences between HBM and FM composition as well as within FM of various brands and in HBM itself (between individual mothers and lactation period). Several metabolites were found exclusively in HBM and FM. Statistically significant higher levels of isoleucine and methionine in their free form were detected in FM samples based on caprine milk, while FM samples based on bovine milk showed a higher level of glucose and galactose in comparison to HBM. The results suggest that the amelioration of FM formulation is imperative to better mimic the composition of minor nutrients in HBM.

## 1. Introduction

Early nutrition has a great influence on a child’s growth and development. Human breast milk (HBM) is recognized as the gold standard for human infant nutrition since it is a source of a wide range of different valuable components that make it the optimal food for newborns. The World Health Organization (WHO) and United Nations Children’s Fund (UNICEF) jointly stated that due to the unique composition of HBM, any other food is not equal to this precious biofluid and they recommended exclusive breastfeeding for six months after birth and continued breastfeeding along with appropriate complementary foods for up to two years of age or beyond [1]. 

Milk is species specific and is recognized as the only food able to satisfy all the nutritional requirements of an infant. Besides the nutritional role, there are numerous known advantages of breastfeeding on infant’s health. Components of HBM protect breastfed infants against inflammation and various infections, contribute to tolerance and suitable response to inflammation, facilitate development [2], enhance development and growth of infant tissues (e.g., epithelial tissue) [3], and aid in the maturation of the gastrointestinal tract [4] and brain development [5]. Beside these benefits, some studies reported that breastfeeding reduces the risk of later development of allergies [6], decreases risk of cardiovascular disease [7] and hypercholesterolemia [8], and contributes to the psychological well-being of the mother and infant [9]. Therefore, children who are breastfed have better health outcomes in comparison with formula-fed infants [10]. Formula-fed babies have different nutritional status, gut microbiota and growth patterns that may contribute to higher risk of later diabetes, obesity and cardiovascular diseases [11]. However, if breastfeeding is precluded, the introduction of commercial formula milk to a newborn’s diet constitutes the best alternative to fulfill the nutritional requirements of infants. In such situations, the composition of infant milk formula can have great consequences for growing offspring. To provide important health benefits, infant formula should closely match the composition, and nutritional and biological function, of HBM. The complete reproduction of HBM composition is not possible due to the dynamic composition of HBM. Although the HBM composition is used as a basic benchmark for designing milk formula compositions, huge differences persist between infant formulas and HBM [11]. 

In the past, formula milk, also known as infant formula or baby formula, was mostly made from appropriately processed bovine milk. From 2013, according to the European Commission Directive [12] FM can also be made from caprine milk. Among FM, first infant formulas (dedicated for the first six months of baby’s life) and follow-on formulas (after six months of infant age) can be differentiated. In European Union countries, the composition of infant formulas has been regulated by the European Commission Directive since 2006 [13]. According to this document, the composition of first infant formula must comply with specific compositional criteria, to fully meet the specific nutritional requirements of the infant in this period of life. Follow-on formulas are intended for further infant nutrition, when complementary foods are introduced, as it is generally advised to avoid the introduction of bovine milk in the diet during the first year [14]. Thus, the specific nutritional requirements of these products are less strict than for infant formulas. Moreover, FM producers can modify the composition of first infant and follow-on formulas and add new ingredients, but only if such supplementation is justified and does not endanger the health of infants and young children. 

Due to disease, medication or personal circumstances, some women tend to reduce, discontinue or even refuse to breastfeed. Thus, raising awareness regarding breastfeeding and its health benefits through the extension of knowledge about the uniqueness of the composition of HBM is advisable. Comparative studies of HBM’s chemical composition with other dairy products may be supportive in achieving this goal. To ensure the maximization of the nutritional and biological value of HBM substitutes and to develop new, improved infant formulas that can better mimic the benefits of HBM, a deep understanding of HBM composition and the differences between the composition of HBM and available infant’s formulas, including minor nutrients, are constantly required. 

Herein, we present a comparative NMR based metabolomic study of HBM and formula milks based on bovine and caprine milk available on Polish markets. To the best of our knowledge, this is the first study that compares the metabolite composition of HBM samples collected by Polish women in a wide range of lactation stages (from 4 to 19 months), using formula milk of seven brands, based on two types of milk, dedicated for children at different ages. Moreover, growing-up formulas dedicated to young children older than 12 months, omitted in the previously published reports, were included in the study in addition to first and follow-on. Therefore, the study provides insight into the differences between FM and HBM samples at molecular level at various lactation stages, which was not reported earlier. 

## 2. Materials and Methods 

### 2.1. Ethical Approval

Ethical approval for this study was granted by The Independent Bioethics Commission for Research, Medical University of Gdańsk, Gdańsk, Poland (NKBBN/**389**/2019).

### 2.2. Reagents and Materials

Methanol, chloroform, disodium hydrogen phosphate and D_2_O containing 0.05% 3-(trimethylsilyl)proponic-2,2,3,3 acid, sodium salt (TSP) were all purchased from Sigma-Aldrich (St. Louis, MO, USA). MilliQ water (Merck Millipore, Darmstadt, Germany) was used for analyses.

### 2.3. Milk Samples

Human breast milk, samples were collected from Polish mothers (*n* = 22) of healthy full-term infants. Study participants were familiarized with the purpose and nature of the study and informed written consent was obtained before participation. To ensure reliable metabolomic results, each breastfeeding woman and her breastfed child included in the research had to meet inclusion criteria which are presented in detail in the Supplementary Materials as the inclusion criteria list. HBM samples were collected from mothers in the various stages of lactation in the range 4 to 19 months. HBM samples were collected during three subsequent days (two samples per day collected, at the same time in the morning and evening), into clean and sanitised milk bottles after full expression from one breast using a milk pump. Approximately 10 mL aliquots were transferred to sterile tubes by donors and stored at −20 °C before shipping to the laboratory for storage at −80 °C until analysis. The remaining amount of HBM was used at the discretion of mothers.

In total, 22 sets of HBM samples were collected (total milk samples *n* = 130). Milk samples were categorized according to the length of time of postpartum into three main groups: under six months (<6 months), between seven and twelve months and older than twelve months (>12 months). The characteristics of the donors of HBM samples are presented in Table S1.

Infant formulas were acquired from a pharmacy. The selection of modified milk was based on the modified milk rankings, which show the most frequently purchased types of FM in Poland (available online) and their origin. Most of the commercial infant formula milk is based on bovine milk, however, alternatives based on caprine milk are also available. The infant formulas with distinct characteristics based on both bovine and caprine milk were included in this study to ensure comprehensive results. Four of the most popular brands of routine infant formula based on bovine milk (A, B, C, D) and three available brands of infant formula based on caprine milk (E, F, G) were chosen for the study. First infant formula and follow-on formulas dedicated to infants between 7 and 12 months and young children older than 12 months were included within each brand. The information regarding FM samples used in the study is presented in Table S2 (Supplementary Materials).

### 2.4. Sample Preparation

Extraction of polar metabolites was based on previously published modified Bligh and Dyer extraction with small modifications [15]. A 225 µL HBM sample was briefly transferred to an Eppendorf microcentrifuge tube and mixed with 950 µL of chloroform/methanol mixture (1/2, *v*/*v*). After 10 s of vigorous vortexing, 310 µL of chloroform and 310 µL of water were added. Subsequently, the sample was vortexed for 10 s, followed by centrifugation at 4 °C for 10 min at 5000× *g*. The 900 µL of aqueous fraction containing polar metabolites was transferred to new centrifuge tube and evaporated to dryness at 30 °C in an Eppendorf Concentrator Plus (Eppendorf Nordic, Hørsholm, Denmark). The evaporated sample was dissolved in 480 µL MilliQ H2O and mixed with 120 µL of phosphate buffer (50 mM Na2HPO4, pH =7.00) and D2O containing 0.05% 3-(trimethylsilyl)proponic-2,2,3,3 acid, sodium salt (TSP; Sigma-Aldrich, St. Louis, MO, USA) mixture (1/1, *v*/*v*). TSP was used as an internal chemical shift reference and for quantification. The obtained HBM metabolite extract was subsequently analyzed using NMR.

### 2.5. NMR Spectroscopy

NMR spectroscopy was performed as described earlier [16]. The 1H NMR spectroscopy was performed, briefly, at 298 K on a Bruker Avance III 600 spectrometer, operating at a 1H frequency of 600.13 MHz, and equipped with a 5-mm 1H TXI probe (Bruker BioSpin, Rheinstetten, Germany). Standard one-dimensional nuclear Overhauser enhancement spectroscopy-presat pulse sequence (noesypr1d) was applied. The following acquisition parameters were used: 64 scans (NS), spectral width (SW) = 7,288 Hz, acquisition time (AQ) = 2.25 sec, 32,768 data points (TD), relaxation delay (D1) = 5 s. All 1H spectra were initially referenced to the TSP signal at 0 ppm. Prior to Fourier transformation, the data were multiplied by a 0.3 Hz line-broadening function. The proton NMR spectra were phase and baseline corrected manually using Topspin 3.0 (Bruker Biospin, Rheinstetten, Germany). NMR signals were assigned in accordance with existing literature [16,17], in-house spectral library, Chenomx NMR Suite 8.1.2 (Chenomx Inc, Edmonton, AB, Canada) and the Human Metabolome Database [18]. Metabolites were quantified using Chenomx NMR Suite 8.1.2 (Chenomx Inc) using known concentration of the internal standard TSP.

### 2.6. Data Analysis

To identify similarities and dissimilarities among the metabolite profile of HBM and FM samples, the principal component analysis (PCA) was performed. The quantitative data were autoscaled prior to analysis by division with the standard deviation. The multivariate data analysis was performed using Latentix 2.13 software (Latentix ApS, Frederiksberg, Denmark). To visualize the qualitative differences between polar metabolite composition of HBM and FM samples, a Venn diagram was applied (a web tool for generating Venn diagrams: bioinformatics.psb.ugent.be). Only metabolites contained in samples of both group (HBM and FM) were included in further multivariate data analysis. Statistical analysis was performed using the Mann–Whitney *U*-test. The *p*-values 0.05 were consider as significant. Samples with relevant metabolites below detection limit were excluded from statistical consideration.

## 3. Results

**Figure 1:**
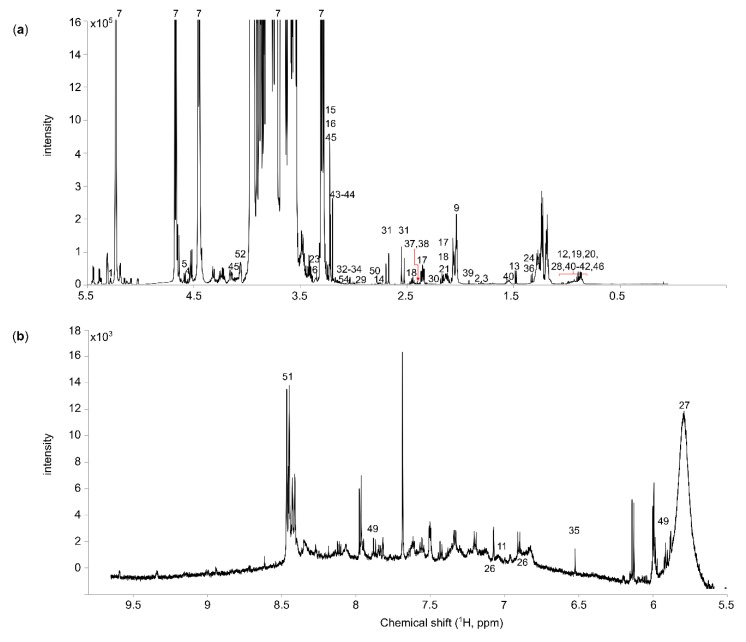
Median 1H NMR spectrum of human breast milk samples (*n* = 130). (**a**) Aliphatic region 5.0–0 ppm; and (**b**) aromatic region 5.5–9.7 ppm.

The collected samples were analyzed using NMR spectroscopy to compare the metabolite profile of HBM with various infant formulas. The representative median spectrum of all analyzed HBM samples is shown in Figure 1. Each signal corresponds to resonances from protons in metabolites according to assignments listed along with their chemical shifts in Table 1. Fifty-four polar metabolites were identified and used in the comparative analysis. The representative median spectrum of all analyzed FM samples is shown in Supplementary Materials on Figure S1.

### 3.1. Comparison of Human Breast Milk Polar Metabolite Composition

The multivariate data analysis was applied to discover similarities and dissimilarities between the metabolite profiles of HBM samples obtained from mothers in various months of lactation. The HBM samples were collected by each woman every day, twice a day (in the morning and in the evening), during the three-day period.

**Figure 2:**
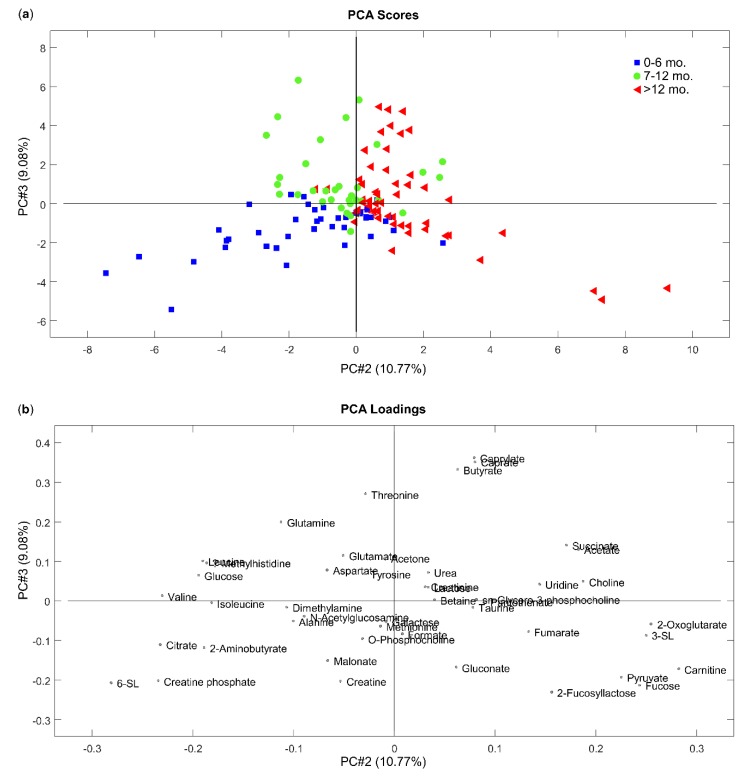
Principal component analysis (PCA) of human breast milk (HBM) samples. (**a**) Scores plot; and (**b**) loading plot.

Initially, a PCA of HBM samples collected in the morning and in the evening was tested. The results indicate no clear difference between those two group of HBM samples (Supplementary Materials Figure S2). Afterwards, we applied the same multivariate data analysis technique to study differences between metabolite profile of HBM samples obtained from different women participating in the study (Figure 2). Due to the nature of the study (comparison of HBM metabolite profile with metabolite profile of infant formula), samples were classified into three groups (Table S1), the same way the infant formulas are classified (first and follow-on formulas). The scores plot (Figure 2a) shows the three distinct groups of HBM corresponding to their lactational age on the plane of the second and third principal components (19.85% of the total variance). Some overlap exists between groups most prominent between the two later groups.

**Figure 3:**
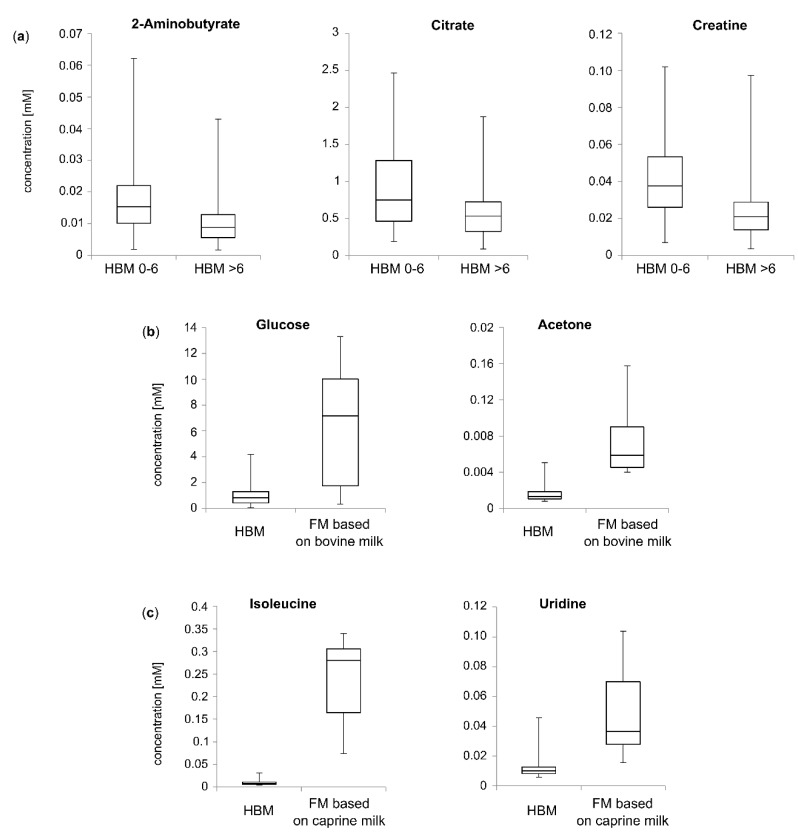
Box plots showing the distribution of polar metabolites indicated as statistically significantly different between analyzed samples: (**a**) 2-aminobutyrate, citrate, creatine in HBM samples; (**b**) glucose and acetone in HBM and formula milk (FM) based on bovine milk samples; and (**c**) isoleucine and uridine in HBM and FM based on caprine milk samples. Horizontal lines indicate medians, boxes specify interquartile range and vertical lines denote the ranges. The comparison was made using the Mann–Whitney *U* test.

Detailed examination of the PCA model showed that HBM samples obtained from participants in their first six months of lactation were located in the lower left part of the PCA scores plot (Figure 2a), separately from HBM samples obtained from other participants who were in subsequent months of lactation. Points corresponding to samples collected from women between 7 and 12 months of lactation tended to group mostly in the middle of the scores plot (between two other groups of samples) and the upper left part of the chart, whereas points representing samples collected after the 12th month of lactation are located in the right part of the scores plot (Figure 2a). An investigation of the metabolites contributing in making this 2D space (Figure 2b) shows that the most discriminant polar metabolites for HBM samples collected in the first six months of lactation were the human milk oligosaccharides (HMO) 6-SL and N-Acetylglucosamine, amino acids and derivatives (2-aminobutyrate, alanine, isoleucine), energy metabolites (citrate, creatine, creatine phosphate) and malonate. The difference in the concentrations of 6-SL, N-Acetylglucosamine, alanine, isoleucine and malonate in HBM samples collected before and after six months of lactation were not statistically significant. Citrate (*p* < 0.001), 2-aminobutyrate (*p* < 0.001), creatine (*p* < 0.001) and creatine phosphate (*p* < 0.001) were found to be present in higher statistically significant levels in HBM samples collected up to the sixth month of lactation compared with HBM samples collected after the sixth month of lactation (Figure 3a). Despite this observation, the small number of participants in this study (small number of participants representing respective groups) prevents us from drawing any conclusions in terms of trends of HBM metabolite composition. Nevertheless, our results show that HBM composition in terms of polar metabolites varies in the different stages of lactation.

A table consisting of quantitative information on polar metabolites in HBM samples can be found in Supplementary Materials Table S3.

### 3.2. Comparison of Formula Milk Polar Metabolite Composition

**Figure 4:**
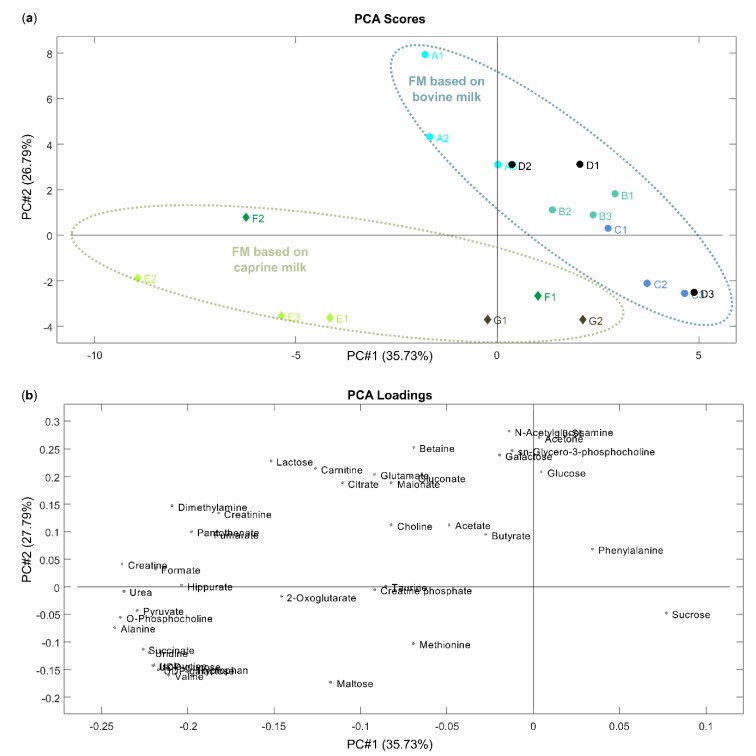
PCA of FM samples. (**a**) Score plot, samples colored for brands (see Table S2); and (**b**) loading plot.

To investigate the differences and similarities between metabolite composition in FM samples, an unsupervised PCA analysis was performed, obtaining a clear discrimination. The obtained 2D PCA scores plot, in which samples are colored according to milk basis (formula milk based on bovine or caprine milk) and the contribution of polar metabolites in making this 2D space, are shown in Figure 4a,b, respectively. The points corresponding to samples based on milk from the same species tended to grouped on the plane of the two first principal components (59.36% of the total explained variance).

The samples of brands based on bovine milk (A, B, C, D) are located in the upper right part of the scores plot, separately from FM samples of brands based on caprine milk (E, F, G), which were clustered in the lower part of the scores plot. This means that compositional differences in the profile of polar metabolites of the FM samples strongly depends on the milk-base used in production. Samples of brands E, F and G have higher levels of some amino acids (alanine, isoleucine, methionine, taurine, tryptophan, valine), nucleotides and derivatives (UDP-galactose, UDP-glucose and uridine), maltose, creatine phosphate and succinate, but have lower levels of acetone, galactose, glucose, N-acetylglucosamine, sucrose, phenylalanine and sn-glycero-3-phosphocholine in comparison to the FM samples based on bovine milk. UDP-galactose, UDP-glucose and valine were detected in all FM samples based on caprine milk (brand E, F, G), whereas in the FM samples based on bovine milk (brand A, B, C, D) these metabolites were below limit of detection. The presence of isoleucine was characteristic of all FM sample based on caprine milk, while it was quantified in only one FM sample based on bovine milk (sample A2, Table S2). The lack of valine and isoleucine in the FM samples based on bovine milk is concerning since these metabolites are essential amino acids, and to match the energy value of HBM, first infant and follow-on formulas need to contain a relevant amount of amino acids, which is set out in the European Commission Directive from 2006 [13]. However, from a quantitative point of view, the amount of the free amino acids in HBM should not be considered alone because substantial differences between the profile of free amino acids and the profile of protein-derived amino acids may exist.

Creatine phosphate was found only in the FM samples based on caprine milk of the brand F. The samples of this brand (brand F), as well as the first infant formula of brand D (D1), were also distinguished by the presence of taurine (not detected in other samples) (Table S4). According to the European Commission Directive from 2006 [13], the amount of taurine in milk formulas must not be greater than 12 mg/100 kcal. The maximum value was not exceeded in these samples (10.13 mg/100 kcal in sample with the highest concentration of taurine (F1, Table S2))

The first infant formula, brand D (sample D1, Table S2), was differentiated by the high concentration (0.746 mM) of phenylalanine (not detected in other samples) (Table S4). The sample of follow-on formula of brand C (dedicated for children older than 12 months, C3) was distinguished from the other formulas by the high concentration level of sucrose, which was consistent with the list of ingredients presented on the label of this FM. Although sucrose was not reported in the ingredients list of other FM samples, this sugar was also detected in the sample of brand A follow-on formula (A3, Table S2), but at a lower concentration level than in sample of follow-on formula of brand C (0.664 mM and 4.961 mM respectively).

A table containing detailed quantitative information on polar metabolites in FM samples can be found in Supplementary Materials Table S4.

### 3.3. Comparative Metabolomics of HBM and FM Samples

**Figure 5:**
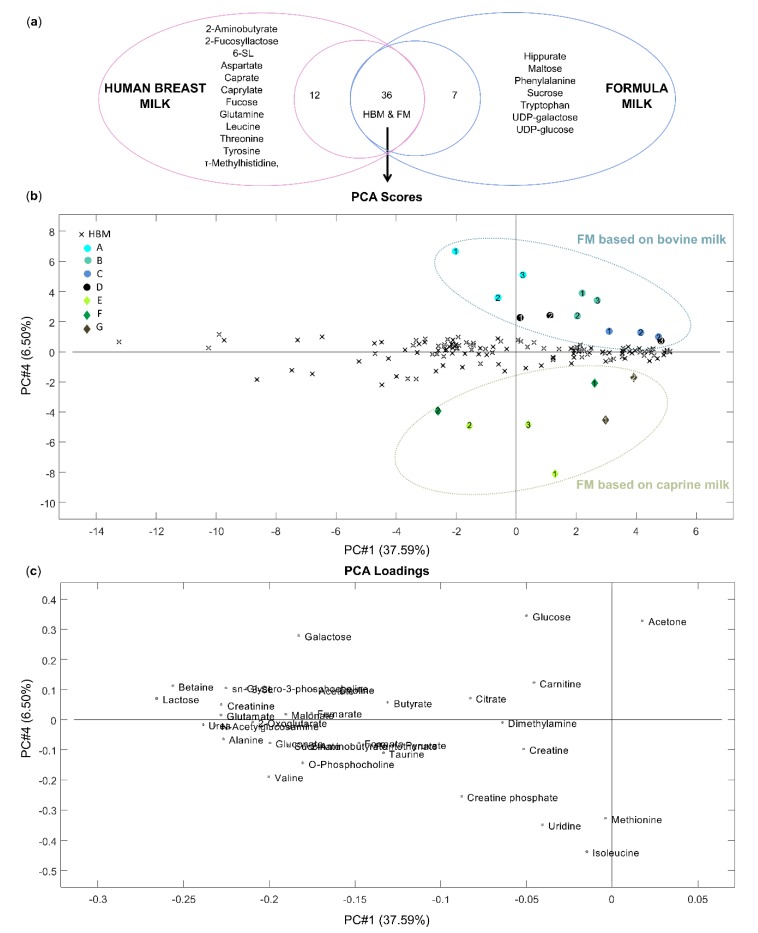
(**a**) Qualitative differences between polar metabolite composition of HBM and FM visualized by a Venn diagram; PCA of HBM and FM samples. (**b**) Score plot, samples colored for HBM and brands of FM (see Table S2); and (**c**) loading plot.

A comparison between the metabolite profile of HBM and FM samples was performed to determine similarities and dissimilarities between these two groups. Initially, qualitative differences between metabolite composition of HBM and FM were visualized using a Venn diagram. The Venn diagram, corresponding to the NMR results of HBM and FM samples (Figure 5a), demonstrated different polar metabolite compositions for each group and the presence of unique metabolites in HBM samples (12) and additional metabolites in FM samples, not detected in HBM sample in our analytical condition (7).

Tryptophan was not detectable in all HBM samples nor almost all of the FM samples based on bovine milk (excluding A1, A2 and B2 samples, which had the mean concentration value of tryptophan of 0.0636 mM), whereas FM samples based on caprine milk contained a mean value of 0.43 mM, with higher values in brand D and lower values in brands F and G. However, as was mentioned above, due to the presence of substantial differences between the profile of free amino acids and the profile of amino acids bound in protein, the concentration level should not be considered alone, since it can lead to a false conclusion.

The disaccharides maltose and sucrose are not secreted in HBM, therefore they were only detected in a few FM samples. Maltose was quantified mostly in follow-on formulas based on bovine milk and in all FM samples based on caprine milk.

To increase the weight of common metabolites in the multivariate models, further comparison between HBM and FM samples was performed, excluding metabolites that were unique for one of group (HBM or FM samples). Metabolites unique to one of group (HBM or FM samples) were removed from further analyses. A PCA of HBM and FM samples was generated using four principal components, which together, explained 68.90% of the total variance described by the PCA model. The obtained 2D PCA scores plot of PC1 and PC4 and the contribution of polar metabolites made up the 2D space shown in Figure 5b,c, respectively.

The scores and loadings plots (Figure 5b,c) clearly show that composition of polar metabolites is different in HBM and FM based on bovine and caprine milk samples. The contribution plot allowed for the identification of the metabolites responsible for the difference between polar metabolite profiles of FM and HBM samples. Infant formulas based on bovine milk (A, B, C, D) were located in the upper right part of the scores plot, separate from other points, because of significantly higher levels of glucose (*p* < 0.001) and acetone (*p* < 0.001) and higher but not statistically significant levels of galactose (Figure 3b). According to the European Commission Directive from 2006 [13], if glucose is added to FM, its content shall not exceed 2 g/100 kcal. Despite high levels of glucose in FM samples based on bovine milk in comparison with HBM samples, the maximum value of this carbohydrate was not exceeded in analyzed samples. However, due to the high concentration of free monosaccharides in FM samples based on bovine milk, the detailed study of their health outcomes in neonates is of primary importance.

For FM samples based on caprine milk (E, F, G), creatine phosphate, uridine, methionine and isoleucine were the metabolites mostly responsible for their clustering in the lower part of the scores plot. Creatine phosphate was found to be present at a higher level only in the FM samples based on caprine milk of the brand F compared with HBM samples, but the concentration difference was not statistically significant. Methionine was found to be present in higher levels only in the first formula samples based on caprine milk of brand E and G (samples E1 and G1) compared with HBM samples. Uridine was quantified in only a few HBM samples at a significantly lower level than in the FM samples based on caprine milk (*p* < 0.001). Isoleucine (*p* < 0.001) was found to be present in significantly higher levels in the FM samples based on caprine milk compared to the HBM samples (Figure 3c).

## 4. Discussion

Results showed that the HBM metabolome varied with the time post-partum, with observed differences between HBM collected up to the sixth, between 7 and 12 and after 12th months of lactation. This observation suggests that the content of polar metabolites in HBM changes during the whole lactation period, and not only during the colostrum and transitional lactation phases. A similar observation was already reported, however this was in regard to non-polar metabolites [19].

Infant formula producers aim to mimic HBM composition in high detail and specific criteria exists for the compositions of starting formulas. Therefore, it was assumed, independent from the source of raw materials, that the typologies (first or follow-on infant formulas) would be the main descriptor of FM metabolite profiles besides the bovine/caprine milk source. However, obtained results showed that compositional similarities come firstly from brand affiliation and not from typology. The observed differences likely result from protein source used for the production of formula milk. Generally, FM based on caprine milk contains pasteurized whole goat milk, while FM based on bovine milk contains bovine whey as a protein source. FM based on caprine milk was differentiated from HBM and other FM samples mostly by its high concentration of amino acids, while FMs based on bovine milk were distinguished from HBM and other FM samples mostly by their high concentration of sugars. Statistically significant higher levels of isoleucine and methionine in their free form were detected in FM samples based on caprine milk, while FM samples based on bovine milk showed a higher level of glucose and galactose in comparison to HBM.

According to the European Commission Directive from 2006 [13], first infant and follow-on formulas must contain an available quantity of amino acids (the sum of free amino acids and amino acids bound in proteins) at least equal to that contained in the indicating reference protein that is HBM. However, free amino acids can be added to milk formulas, only to improve the protein nutritional value, and solely in the proportion essential for that purpose. In HBM, the quantity of free amino acids guarantees approximately 3%–5% of the infant demand for those compounds. The remaining 97%–95% is provided by protein intake [20]. Since the distribution and organization of each HBM component is not assumed to be accidental, the proportion of free amino acids to protein-derived amino acids may also be of importance. To release the amino acids bound in protein, proteins require the proteolytic activity of the digestive tract. Free amino acids undergo absorption more readily than amino acids bound in protein (proteolysis is not required), therefore they reach the pool of endogenous amino acids much earlier than protein-derived amino acids. Due to the distinct absorption time lag, a difference in metabolism and physiological utilization may occur. The amino acid imbalance at the metabolism sites after absorption of the free amino acids leads to the lower utilization of free amino acids. This produces the catabolism of the free amino acid’s surplus [21].

Theoretically, the significantly higher level of isoleucine in FM should not have any impact on infant health, since high doses of dietary branched amino acids are well tolerated by the human body (due to the reserve of branched-chain α-ketoacid dehydrogenase complex activity present in body tissues). The safe upper limit of dietary branch amino acids in general (over which the rate of branched-chain α-ketoacid dehydrogenase complex is limited), has not been established so far. However, the toxicity of leucine, when it is consumed out of proportion to isoleucine and valine should be kept in mind [22]. Therefore, the high concentration of isoleucine in its free form in FM samples is insignificant, as long as a suitable ratio of branch amino acids is sustained. The significantly higher level of methionine in its free form is questionable, however, due to the risk of non-genetic hypermethioninemias that might be caused by the ingestion of relatively high amounts of this amino acid [23,24].

A high concentration of phenylalanine (not detectable in all HBM samples) in the first infant formula based on bovine milk of brand D (sample D1) is also questionable, since it poses the question of the risk of accumulation in the blood and tissues of children with phenylketonuria [25]. The gold standard treatment for patients with phenylketonuria is a low phenylalanine diet. The amount of phenylalanine that a person with phenylketonuria can consume depends on age, body weight and individual tolerance to phenylalanine [26]. The list of ingredients presented on the label of the first infant formula of brand D only includes information about the presence of phenylalanine, without information about a concentration level. According to the European guidelines, the level of phenylalanine in the blood of infants with phenylketonuria should be maintained between 120 and 360 μmol/L. For this reason, the phenylalanine intake is decreased and titrated through combination of breastfeeding or feeding with standard FM with phenylalanine-free infant formula [27]. Therefore, information about the presence of phenylalanine on the label of the first infant formula of brand D seems to be insufficient.

Moreover, despite lower concentration levels of galactose and glucose in FM based on caprine milk compared with HBM (not statistically significant), their presence, together with the high amount of free amino acids, can lead to a range of off-flavors and off-odors and various degrees of browning (Maillard reaction) if the product is stored incorrectly [28].

Our results show qualitative and quantitative differences between HBM and FM composition as well as within the FM of various brands and in HBM itself, which is consistent with other studies [15,16,29,30,31]. Metabolite profiles of FM samples were distinct between studied brands. We did not observe significant differences with respect to target age range. A comparative study of metabolite profiles of FM purchased on Italian markets based on skimmed bovine milk and HBM from Italian women reported similar conclusions, based, however, on other differences between metabolites profiles of FM and HBM [30]. The most discriminant metabolites of FM purchased on Italian markets based on bovine milk were galactose (also one of the most discriminant metabolites for FM based on bovine milk purchased in Polish pharmacy), and mannitol (not detected in the present study) and malic acid that was not reported in the present study. Italian HBM contained higher levels of amino acids, urea and inositols than Italian FM. In our study, higher levels of amino acids were characteristic for FM based on caprine milk that was not included in the study by Scano et al. (2016) [30]. The variances between detected differences might come from different compositions of FM purchased on Italian and Polish markets and breast milk metabolite composition variation by ethnicity or geographical location [32,33].

To sum up, the results of multivariate analysis of NMR data of polar metabolite composition indicate that the polar metabolite profile of HBM samples varied among the stages of lactation. The grouping of HBM samples according to the month of lactation was observed. Despite great efforts of dairy-based formula producers to mimic HBM composition, similar grouping was not observed among FM samples. The compositional similarities in formula milk samples came from brand affiliation not from typologies (first or follow-on infant formulas). FMs based on bovine milk are distinguished from HBM and other FM samples mostly by high concentration of sugars, and FM based on caprine milk are differentiated from HBM and other FM samples mostly by a high concentration of amino acids.

Our results show that FM samples differentiate from HBM and among themselves, and therefore can have different outcomes in neonatal nutrition. Unlike HBM, whose composition is dynamic, and varies over lactation, the first infant formulas and follow-on formulas are standardized within the range of compositions. As milk formulas are sophisticated food products that are specially produced for an intended purpose, the directives in regard to their production are quite strict, but are still in the field of major nutrients. The guidelines on the composition of minor nutrients are not so rigorous. The knowledge about the composition of low molecular compounds such as polar metabolites contained in HBM is still poor. However, biological functions of HBM polar metabolites such as shaping the infant’s intestinal microbiota, modulation of the immune system, neurological health benefits or facilitating metabolism of lipids have been proven [34,35,36,37,38,39,40]. Thus, advancements on the knowledge of the composition of HBM polar metabolites and the amelioration of FM compositions in the range of their compounds is of great importance regarding optimal infant nutrition. Moreover, the necessity of the amelioration of FM compositions is of critical importance, especially in the light of the results of nutritional studies on infants carried out comparing HBM to FM feeding, which indicate different health outcomes for infants who are breastfed compared with those who are fed FM [10]. Due to the diversity of FM compositions, specific FM may have different outcomes in neonatal nutrition. A deep understanding of the composition of HBM serves as a valuable tool for infant feeding management.

## Figures and Tables

**Table 1:** List of identified metabolites with chemical shifts in ppm from internal sodium salt (TSP) standard and assignment resonances.

#	Metabolite	1H Chemical shift (ppm)	Assignment	#	Metabolite	1H Chemical shift (ppm)	Assignment
	**Sugars**				**Energy metabolites**		
1	2-Fucosyllactose	4.22	Fuc(α1-2) CH-5	29	2-Oxoglutarate	2.99	CH_2_
		5.30	Fuc(α1-2) CH-1				
		1.22	Gal(β1-4) CH_3_-6				
		4.52	Gal(β1-4) CH-1				
2	3-SL	1.79	CH_3_	30	Acetone	2.22	2CH_3_
3	6-SL	1.73	CH_3_	31	Citrate	2.53	α-CH_2_
						2.66	α’-CH_2_
4	Fucose	1.19–5.19	Multiple	32	Creatine	3.02	CH_3_
5	Galactose	4.55	CH	33	Creatine phosphate	3.03	CH_3_
6	Glucose	3.23	CH-3	34	Creatinine	3.03	CH_3_
		3.39	CH-5				
7	Lactose	3.28, 3.5–4.0	Multiple	35	Fumarate	6.51	CH=CH
8	Maltose	3.27–5.40	Multiple	36	Lactate	1.32	CH_3_
						4.10	CH
9	N-Acetylglucosamine	2.02	CH_3_	37	Pyruvate	2.36	CH_3_
10	Sucrose	3.46–5.40	Multiple	38	Succinate	2.39	2CH_2_
	**Amino acids and derivatives**				**Fatty acids and associated metabolites**		
11	τ-Methylhistidine	7.06	CH-4	39	Acetate	1.91	CH_3_
12	2-Aminobutyrate	0.97	γ-CH_3_	40	Butyrate	0.88	CH_3_
		1.89	β-CH_2_			1.55	β-CH_2_
13	Alanine	1.47	CH_3_	41	Caprate	0.85	CH_3_
						1.53	CH_2_
14	Aspartate	2.67	CH	42	Caprylate	0.85	CH_3_
		2.80	CH_2_			1.53	CH_2_
15	Betaine	3.26	CH_3_	43	Choline	3.19	3CH_3_
16	Carnitine	2.43	CH_2_	44	sn-Glycero-3-phosphocholine	3.21	3CH_3_
		3.21	3CH_3_				
17	Glutamate	2.12	β-CH_2_	45	O-Phosphocholine	3.21	3CH_3_
		2.33	γ-CH_2_			4.16	CH_2_
18	Glutamine	2.11	β-CH_2_		**Vitamins**		
		2.46	α-CH_2_				
19	Isoleucine	0.93	δ-CH_3_	46	Pantothenate	0.92	CH_3_
		1.00	β-CH_3_				
		1.25	γ-CH_2_				
		1.46	γ’-CH_2_				
		1.97	β-CH				
20	Leucine	0.94	CH_3_		**Nucleotides and derivatives**		
21	Methionine	2.13	CH_3_	47	UDP-galactose^1^	3.72–7.94	Multiple
22	Phenylalanine	3.19	CH_2_	48	UDP-glucose^2^	3.46–7.94	Multiple
		7.32	CH-2,6				
		7.37	CH-4				
		7.42	CH-3,5				
23	Taurine	3.25	CH-5	49	Uridine	5.89	CH-2
		3.42	CH-6			5.9	CH-10
						7.86	CH-11
24	Threonine	1.31	γ-CH_3_		**Others**		
				50	Dimethylamine	2.71	2CH_3_
25	Tryptophan	7.19	CH-8	51	Formate	8.44	CH
		7.27	CH-7				
		7.31	CH-2				
		7.53	CH-6				
		7.73	CH-9	52	Gluconate	4.05	CH
26	Tyrosine	6.89	CH-3,5	53	Hippurate	7.63	CH-4
		7.18	CH-2,6			7.82	CH-3,5
27	Urea	5.78	NH_2_	54	Malonate	3.12	CH_2_
28	Valine	0.98	γ-CH_3_				
		1.03	γ’-CH_3_				

^1^ Uridine diphosphate galactose. ^2^ Uridine diphosphate glucose.

## Supplementary Materials

The following are available online at https://www.mdpi.com/2072-6643/12/4/921/s1, inclusion criteria for the HBM samples donors, Figure S1: Median ^1^H NMR spectrum of formula milk samples (*n* = 19). (a) Aliphatic region 5.0–0 ppm; and (b) aromatic region 5.5–9.7 ppm, Figure S2: 2D score plot of PCA analysis showing no difference between morning and evening HBM samples, Table S1: Characteristics of HBM sample donors, Table S2: Characteristics of FM samples, Table S3: Polar metabolite concentration determined in human breast milk samples, Table S4: Polar metabolite concentration determined in formula milk samples.
