# Supplementary material for: Nuclear Magnetic Resonance Metabolomics Reveals Qualitative and Quantitative Differences in the Composition of Human Breast Milk and Milk Formulas
Source: Nutrients. 2020 Mar 27;12(4):921. doi: 10.3390/nu12040921 (PMC7230615; doi:10.3390/nu12040921)
Supplement: Supplementary file 1 [file nutrients-12-00921-s001.pdf]

nutrients

MDPI

Article

Nuclear Magnetic Resonance Metabolomics Reveals Qualitative and Quantitative Differences in the Composition of Human Breast Milk and Milk Formulas

Dorota Garwolińska [1,2,*], Weronika Hewelt-Belka [1], Agata Kot-Wasik [1] and Ulrik Kræmer Sundekilde [2]

[1] Department of Analytical Chemistry, Faculty of Chemistry, Gdańsk University of Technology, Gabriela Narutowicza 11/12, 80-233 Gdańsk, Poland; weronika.belka@pg.edu.pl (W.H.-B.); agawasik@pg.edu.pl (A.K.-W.)

[2] Department of Food Science, Faculty of Technical Sciences, Aarhus University, Agro Food Park 48, DK-8200 Aarhus N, Denmark; uksundekilde@food.au.dk

* Correspondence: dorota.garwolinska@pg.edu.pl

Received: 4 March 2020; Accepted: 25 March 2020; Published: date

Inclusion criteria for the HBM samples donors

Women and their children participating in the study had to meet the inclusion criteria: exclusively/fully lactating mothers and their breastfeeding singleton infants living in Pomeranian Voivodship, Poland were eligible for inclusion in the study if they continued full breastfeeding for the first 6 months. Used of complementary foods starting at 6 months was permitted for all infants as per American Academy of Pediatrics (AAP) guidelines and World Health Organization recommendations as long as their sole milk source was maternal breast milk. Infants had to be ≥35 weeks' gestation and in good general health at the time of enrollment. Subject exclusion criteria will include: mother's diagnose of preexisting type I or II diabetes, hypertension, parathyroid disease, and uncontrolled thyroid disease. Women of twins or multiple births were not eligible to participate in the study. Infants 35 weeks' gestation; with a history of 72 hours in the NICU; any inborn error of metabolism; history of congenital anomalies; or a history of consuming 10% of their diet as formula at the time of enrollment were not eligible for participation in the study. Twins and multiples were not eligible for participation in the study. Women who were combination feeders at the time of enrollment (i.e., partially breastfeeding and formula-feeding their infants) were not eligible to participate in the study.

Mothers were also asked to complete a survey on the state of their health and health of their child, lifestyle, diet, child/mother infections, supplementation with vitamin D3 and DHA by the mother, maternal diet rich/poor in unsaturated fatty acids.

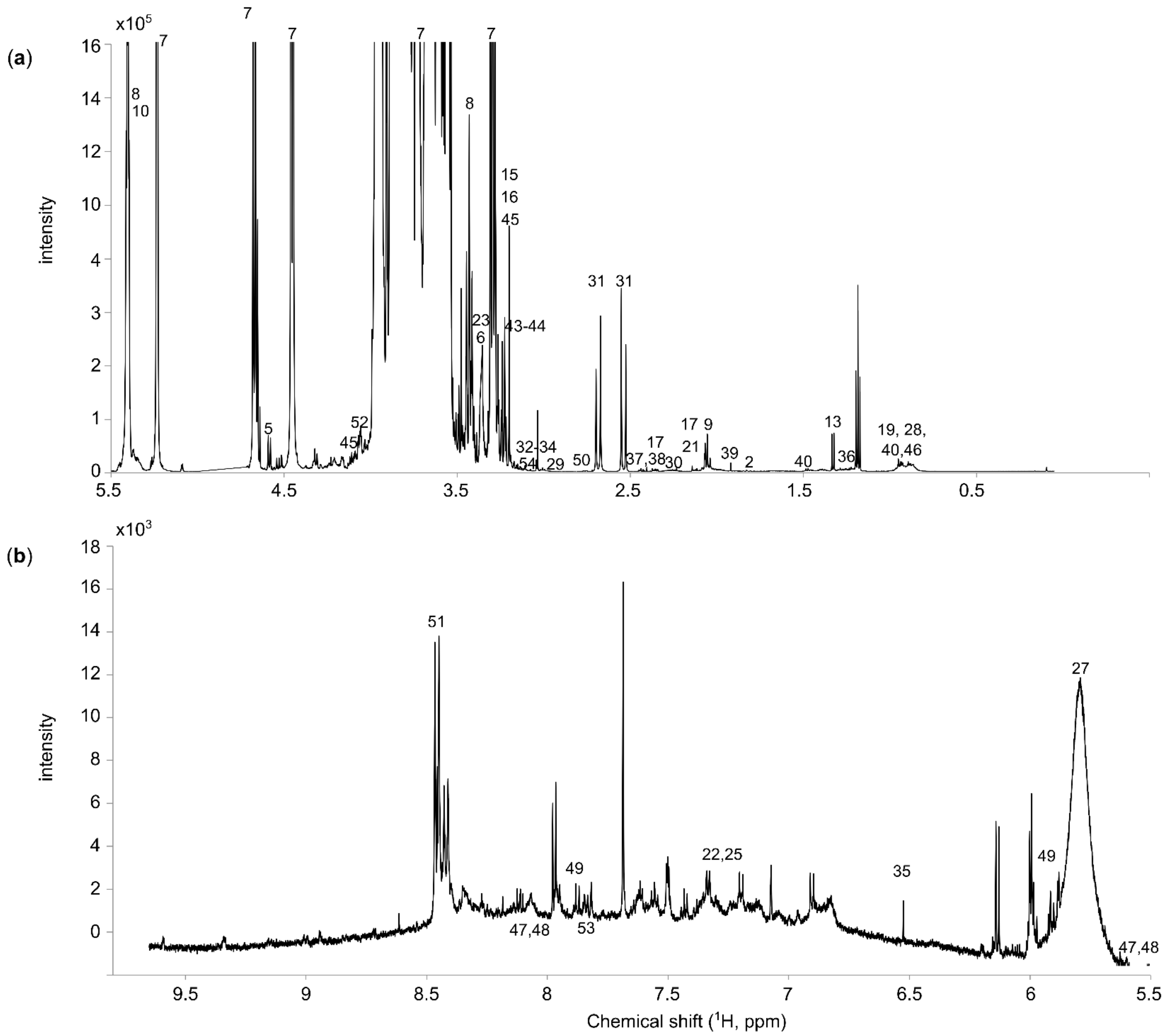

Figure S1. Median 1H NMR spectrum of the formula milk samples (n = 19): (a) Aliphatic region 5.0 – 0 ppm; and (b) aromatic region 5.5 – 9.7 ppm.

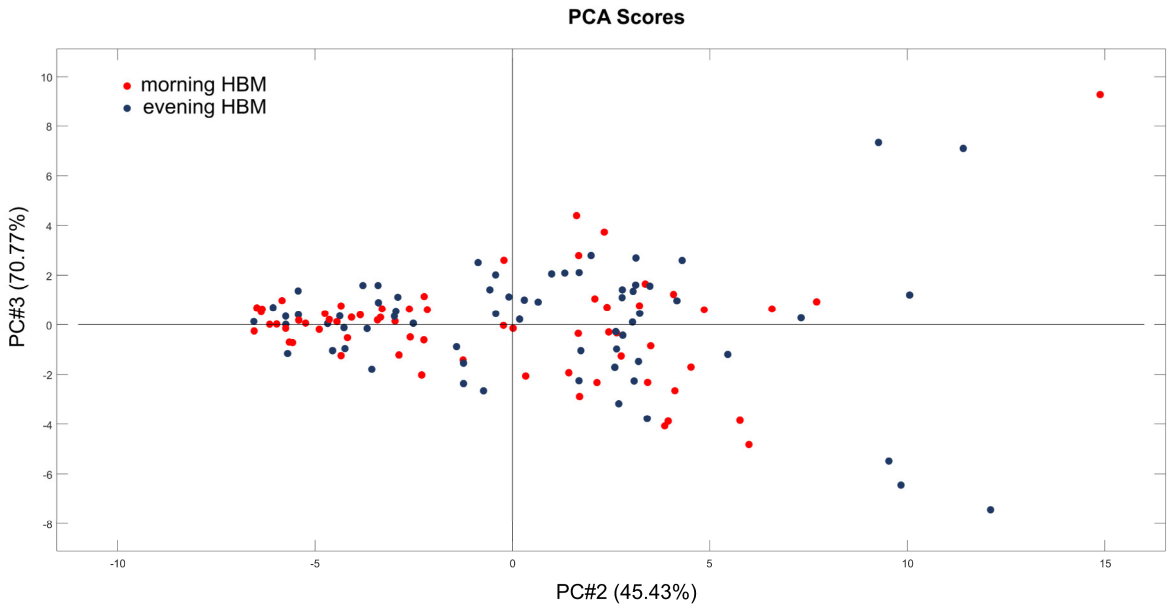

Figure S2. The 2D score plot of the PCA analysis showing no difference between morning and evening HBM samples.

Table S1. Characteristics of the HBM sample donors.

No.	Sample ID	HBM group	Month of Lactation	Gestation	Age of Mother, Years
1	4A	0-6	4	39	35
2	4B		4	40	29
3	5		5	40	29
4	6A		6	39	35
5	6B		6	39	35
6	6C		6	40	29
7	6D		6	41	30
8	7A	7-12	7	41	26
9	7B		7	39	29
10	8A		8	41	26
11	8B		8	39	31
12	11		11	40	29
13	10		10	39	31
14	13A	>12	13	41	27
15	13B		13	40	29
16	15A		15	41	27
17	15B		15	40	29
18	14		14	41	29
19	15C		15	41	28
20	16		16	41	29
21	18		18	41	27
22	19		19	40	28

Table S2. Characteristics of the FM samples.

**Formula Base**	**Bovine's Milk**					**Caprine Milk**	
**Typology**	**Brand**						
	**A**	**B**	**C**	**D**	**E**	**F**	**G**
<6	A1	B1	C1	D1	E1	F1	G1
7–12	A2	B2	C2	D2	E2	F2*	G2*
>12	A3	B3	C3	D3	E3		

*only first and follow on formula dedicated for infants older than 6 months were available.

Table S3. Polar metabolite concentration in human breast milk samples (HBM samples collected in the morning were marked with letter M; HBM samples collected in the evening were marked with letter E; blank boxes mean that relevant metabolite was not detected in the corresponding sample). Polar metabolites concentrations [mM].

ID	2-Aminobutyrate	2-Fucosyllactose	2-Oxoglutarate	3-SL	6-SL	Acetate	Acetone	Alanine
4A_1M	0.0128	2.0680	0.0291	0.0096	0.0237	0.0144		0.3349
4A_2M	0.0368	2.1104	0.0243	0.0117	0.0224	0.0157		0.2909
4A_3M	0.0173	0.9472	0.0136	0.0048	0.0117	0.0117	0.0008	0.1277
4A_1E	0.0101	0.3405	0.0067		0.0064	0.0037		0.0717
4A_2E	0.0221	1.1624	0.0192	0.0075	0.0163	0.0117		0.1813
4A_3E	0.0059	0.6659	0.0120	0.0035	0.0112	0.0075		0.1605
4B_1M	0.0277	2.2685	0.0355	0.0171	0.0077	0.0155	0.0013	0.1211
4B_2M	0.0208	2.6816	0.0485	0.0251		0.0213		0.1421
4B_3M	0.0152	0.9003	0.0157	0.0053	0.0045	0.0083	0.0013	0.0568
4B_1E	0.0040	0.4843	0.0139	0.0037		0.0056		0.0539
4B_2E	0.0067	1.7587	0.0485	0.0123	0.0045	0.0163	0.0019	0.1883
4B_3E	0.0019	0.3333	0.0085	0.0027		0.0056	0.0008	0.0376
5_1M	0.0136	1.0563	0.0251	0.0099		0.0147	0.0013	0.0864
5_2M	0.0144	0.9517	0.0309	0.0091		0.0123	0.0008	0.0784
5_3M	0.0096	1.0619	0.0227	0.0096		0.0101	0.0000	0.0776
5_1E	0.0029	0.5029	0.0131	0.0043		0.0059	0.0008	0.0432
5_2E	0.0080	1.6909	0.0357	0.0144		0.0152	0.0013	0.1285
5_3E	0.0037	0.9741	0.0264	0.0072		0.0120	0.0019	0.0968
6A_1M	0.0088	0.4125	0.0096	0.0000	0.0040	0.0045		0.0664
6A_2M	0.0189	1.4760	0.0264	0.0088	0.0171	0.0112		0.2200
6A_3M	0.0123	0.7224		0.0035	0.0059	2.6667		0.1043
6A_1E	0.0621	2.5152	0.0501	0.0163	0.0349	0.0221		0.4819
6A_2E	0.0469	2.5125	0.0445	0.0163	0.0387	0.0301		0.5771
6A_3E	0.0285	1.3003		0.0064	0.0101	0.0115		0.2448
6B_1M	0.0165	1.5728	0.0280	0.0101	0.0152	0.0136		0.2309
6B_2M	0.0411	1.9205	0.0344	0.0120	0.0149	0.0179	0.0016	0.3197
6B_3M	0.0387	1.5885	0.0397	0.0120	0.0149	0.0149	0.0021	0.2760
6B_1E	0.0101	0.5165	0.0139	0.0027	0.0061	0.0069		0.0869
6B_2E	0.0283	1.4883	0.0373	0.0107	0.0229	0.0232		0.4264
6B_3E	0.0304	1.9637	0.0584	0.0149	0.0272	0.0179		0.6405
6C_4M	0.0083	0.6555	0.0168	0.0069		0.0080	0.0008	0.0587
6C_2M	0.0131	1.9213	0.0472	0.0224		0.0187	0.0029	0.1931
6C_3M	0.0104	0.8200	0.0277	0.0083		0.0080	0.0000	0.0592
6C_1E	0.0216	1.6784	0.0491	0.0203		0.0205	0.0019	0.1392
6C_2E	0.0104	1.0235	0.0227	0.0101		0.0096	0.0011	0.0645
6C_3E	0.0155	2.4667	0.0600	0.0269		0.0261	0.0019	0.2283
6D_1M	0.0203	0.8112	0.0187	0.0075		0.0059		0.0963
6D_2M	0.0203	0.6672	0.0083	0.0093		0.0077		0.1037
6D_3M	0.0240	1.1480	0.0240	0.0157	0.0085	0.0117	0.0021	0.1445
6D_1E	0.0128	0.4688	0.0085	0.0045		0.0059		0.0736
6D_2E	0.0195	0.7192	0.0152	0.0080		0.0109		0.1525

6D_3E	0.0171	1.1715	0.0163	0.0152	0.0040	0.0128		0.1912
7A_1M	0.0085	1.2691	0.0448	0.0157	0.0237	0.0123	0.0021	0.0965
7A_2M	0.0032	0.3573	0.0139	0.0056	0.0072	0.0045	0.0011	0.0341
7A_3M	0.0029	0.2325	0.0099	0.0027	0.0056	0.0029	0.0013	0.0147
7A_1E	0.0093	1.3560	0.0347	0.0189	0.0235	0.0160	0.0016	0.2043
7A_2E	0.0029	0.5053	0.0179	0.0067	0.0115	0.0091		0.0949
7A_3E	0.0059	0.4856	0.0176	0.0080	0.0072	0.0064	0.0011	0.0600
7B_1M	0.0067	0.9717	0.0141	0.0075		0.0123		0.1051
7B_2M	0.0061	1.1296	0.0109	0.0083	0.0048	0.0141	0.0011	0.1179
7B_3M	0.0117	1.8347	0.0259	0.0173	0.0093	0.0232	0.0008	0.2264
7B_1E	0.0069	0.7829	0.0152	0.0067	0.0029	0.0096		0.1155
7B_2E	0.0123	1.0597	0.0227	0.0088	0.0053	0.0125	0.0013	0.1472
7B_3E	0.0331	1.9107	0.0381	0.0155	0.0093	0.0221	0.0008	0.2480
8A_2M	0.0016	0.2677	0.0165	0.0040		0.0075		0.0384
8A_3M	0.0080	1.1600	0.0637	0.0147	0.0053	0.0288	0.0013	0.1661
8A_1E	0.0075	1.1600	0.0443	0.0141	0.0061	0.0269	0.0008	0.1592
8A_3E	0.0037	1.1288	0.0627	0.0128	0.0053	0.0280	0.0013	0.1445
8B_1M	0.0053		0.0099	0.0000		0.0056	0.0013	0.0421
8B_2M	0.0168		0.0237	0.0144	0.0051	0.0141	0.0011	0.1552
8B_3M	0.0021		0.0112	0.0059		0.0165	0.0013	0.0384
8B_1E	0.0179		0.0520	0.0117	0.0069	0.0125	0.0024	0.1971
8B_2E	0.0080		0.0317	0.0168	0.0043	0.0173	0.0019	0.1568
8B_3E	0.0123		0.0368	0.0136		0.0131		0.1565
10_1M	0.0269		0.0304	0.0120		0.0232	0.0051	0.1699
10_2M	0.0429		0.0259	0.0147		0.0211	0.0016	0.2309
10_3M	0.0192		0.0301	0.0120		0.0413	0.0016	0.2152
10_1E	0.0171		0.0235	0.0128		0.0205		0.2096
10_2E	0.0064		0.0523	0.0101		0.0664	0.0011	0.2989
10_3E	0.0077		0.0451	0.0109		0.0291	0.0011	0.2701
11_4M	0.0243		0.0488	0.0299		0.0320		0.2632
11_2M	0.0045		0.0141	0.0088		0.0069	0.0011	0.0451
11_3M	0.0053		0.0104	0.0064		0.0075	0.0019	0.0259
11_1E	0.0109		0.0608	0.0267		0.0163	0.0029	0.2373
11_2E	0.0104		0.0280	0.0123		0.0104	0.0011	0.1197
11_3E	0.0061		0.0413	0.0227		0.0176	0.0013	0.1336
13A_1M	0.0040	0.5451	0.0267	0.0051		0.0109	0.0013	0.0523
13A_2M	0.0085	1.2421	0.0403	0.0125		0.0309		0.1944
13A_3M	0.0117	1.2208	0.0373	0.0152		0.0299	0.0019	0.1688
13A_1E	0.0096	1.5365	0.0592	0.0115		0.0349	0.0008	0.2784
13A_2E	0.0072	1.5144	0.0685	0.0131		0.0307	0.0011	0.2339
13A_3E	0.0099	1.2048	0.0507	0.0149		0.0408		0.2315
13B_1M	0.0101	0.4341	0.0141	0.0133		0.0136	0.0013	0.0795
13B_2M	0.0165	0.2277	0.0176	0.0048		0.0072		0.0496
13B_3M	0.0355	0.9605	0.0403	0.0277		0.0205		0.1912
13B_1E	0.0256	1.1184	0.0493	0.0360		0.0403		0.3211
13B_2E	0.0277	1.1112	0.0440	0.0347		0.0224		0.3117

13B_3E	0.0104	0.4331	0.0195	0.0125		0.0107		0.1184
14_1M	0.0208	3.3176	0.0739	0.0144		0.0304		0.1605
14_2M	0.0187	2.1653	0.0184	0.0091		0.0168	0.0016	0.1096
14_3M	0.0035	0.5325	0.0133	0.0021		0.0067		0.0352
14_1E	0.0056	0.8883	0.0264	0.0043		0.0131		0.0645
14_2E	0.0128	2.8875	0.0576	0.0147		0.0235		0.2229
14_3E	0.0064	2.6645	0.0675	0.0112		0.0421		0.1917
15A_1M	0.0237	1.8312	0.0893	0.0229		0.0541		0.2552
15A_2M	0.0067	0.5515	0.0237	0.0037		0.0091		0.0541
15A_3M	0.0088	1.5979	0.0709	0.0141		0.0259	0.0035	0.1547
15A_1E	0.0088	1.6677	0.0331	0.0157		0.0275		0.2373
15A_2E	0.0045	1.5184	0.0523	0.0149		0.0323		0.2136
15A_3E	0.0021	0.3704	0.0221	0.0048		0.0075	0.0008	0.0667
15B_1M	0.0243	1.3200	0.0531	0.0355		0.0229	0.0013	0.2019
15B_2M	0.0048	0.4451	0.0211	0.0123		0.0085		0.0581
15B_3M	0.0091	0.5227	0.0293	0.0128		0.0155	0.0011	0.0952
15B_1E	0.0219	2.0704	0.0877	0.0509		0.0429		0.3773
15B_2E	0.0088	0.9427	0.0509	0.0195		0.0155		0.1779
15B_3E	0.0040	0.8139	0.0368	0.0245		0.0251		0.1541
15C_1M	0.0080	2.6789	0.0835	0.0357		0.0307		0.1469
15C_2M	0.0320	6.3960	0.2061	0.0808		0.0747		0.4176
15C_3M	0.0045	0.6931	0.0251	0.0115		0.0085		0.0357
15C_1E	0.0211	5.3315	0.1256	0.0731		0.0453		0.3611
15C_2E	0.0027	0.8693	0.0200	0.0131		0.0080		0.0571
15C_3E	0.0304	5.1013	0.1485	0.0709		0.0464		0.4461
16_1M	0.0024	0.4613	0.0176	0.0048		0.0056		0.0069
16_2M	0.0091	2.3669	0.0539	0.0237		0.0253		0.0520
16_3M	0.0080	1.2835	0.0347	0.0128		0.0115		0.0864
16_1E		0.9485	0.0309	0.0104		0.0080		0.0453
16_2E		0.4531	0.0203	0.0048		0.0061		0.0232
16_3E	0.0032	1.1373	0.0267	0.0107		0.0123		0.1101
18_1M		0.2603	0.0160	0.0043		0.0075	0.0016	0.0253
18_2M		0.3037	0.0171	0.0040		0.0064	0.0016	0.0240
18_3M	0.0061	1.3749	0.0848	0.0211	0.0091	0.0245	0.0021	0.1408
18_1E	0.0109	1.2861	0.0587	0.0208	0.0051	0.0213	0.0019	0.1992
18_2E	0.0072	0.6261	0.0413	0.0101		0.0120		0.1312
18_3E	0.0109	1.6867	0.0549	0.0304		0.0256	0.0027	0.1989
19_1M	0.0093	0.2899	0.0360	0.0200		0.0216	0.0021	0.1112
19_2M	0.0155	0.6107	0.0259	0.0384		0.0392		0.1955
19_3M	0.0069	0.2629	0.0195	0.0133		0.0107		0.0592
19_1E	0.0088		0.0387	0.0413		0.0240	0.0011	0.1456
19_2E	0.0048	0.4728	0.0379	0.0301		0.0307	0.0008	0.1565
19_3E	0.0088	0.4808	0.0365	0.0341		0.0269		0.1211

ID	Aspartate	Betaine	Butyrate	Caprate	Caprylate	Carnitine	Choline	Citrate
4A_1M	0.0624	0.0725	0.0363	0.0800	0.1109		0.1067	1.6587
4A_2M	0.0611	0.0869	0.0640	0.0704	0.0619		0.1091	2.4613

4A_3M	0.0432	0.0315	0.0461	0.0363	0.0768		0.0555	1.1797
4A_1E		0.0165	0.0072	0.0125	0.0179		0.0293	0.4363
4A_2E	0.0477	0.0371	0.0115	0.0112	0.0093		0.0787	1.1072
4A_3E	0.0363	0.0227	0.0256	0.0461	0.0627		0.0411	0.6027
4B_1M	0.0291	0.0680	0.0979	0.0629	0.1795		0.2856	1.5477
4B_2M		0.0784	0.0563	0.0291	0.0309		0.2861	1.8203
4B_3M		0.0248	0.0291	0.0179	0.0520		0.1323	0.7208
4B_1E	0.0299	0.0195	0.0323	0.0315	0.0573	0.0112	0.0664	0.3229
4B_2E	0.0325	0.0589	0.0195	0.0331	0.0461		0.2232	0.8624
4B_3E		0.0125	0.0120	0.0069	0.0240	0.0093	0.0453	0.1955
5_1M	0.0376	0.0421	0.0779	0.1056	0.2325		0.1763	0.5395
5_2M	0.0203	0.0376	0.0291	0.0424	0.0517		0.1851	0.6096
5_3M	0.0248	0.0320	0.0443	0.0520	0.0992		0.2139	0.4621
5_1E		0.0128	0.0219	0.0451	0.0811		0.0933	0.1845
5_2E	0.0480	0.0464	0.0376	0.0579	0.0952		0.3528	0.7928
5_3E	0.0323	0.0331	0.0312	0.0571	0.0845		0.2205	0.4117
6A_1M		0.0168		0.0157	0.0216		0.0352	0.4528
6A_2M	0.0440	0.0368	0.0227	0.0208	0.0365		0.1152	1.1939
6A_3M		0.0277	0.0155	0.0109	0.0205		0.0395	0.6493
6A_1E	0.1723	0.0787		0.0208	0.0328		0.3221	2.0997
6A_2E	0.1683	0.1133	0.0781	0.0853	0.1000		0.2365	2.1032
6A_3E		0.0475	0.0269	0.0227	0.0331		0.1304	0.9955
6B_1M	0.0379	0.0685	0.1112	0.0856	0.2493		0.1035	1.3597
6B_2M	0.1008	0.0896	0.0821	0.0547	0.0669		0.1259	1.8117
6B_3M	0.0803	0.0600	0.0848	0.0440	0.0600		0.1685	1.3523
6B_1E		0.0221	0.0251	0.0312	0.0795		0.0552	0.4563
6B_2E	0.1419	0.0533	0.0800	0.0680	0.1088		0.2000	1.3107
6B_3E	0.2021	0.0957	0.0877	0.0635	0.0709		0.2309	2.2637
6C_4M		0.0264	0.0400	0.0435	0.0835	0.0277	0.1344	0.3739
6C_2M	0.0976	0.0688	0.1032	0.0733	0.1032		0.3083	0.8381
6C_3M		0.0339	0.0443	0.0379	0.0648		0.1389	0.4520
6C_1E		0.0499	0.0760	0.0539	0.1133	0.0245	0.2544	0.6931
6C_2E		0.0323	0.0475	0.0499	0.0867	0.0363	0.1392	0.4448
6C_3E	0.0757	0.0920	0.0667	0.0645	0.0971	0.0336	0.4560	1.1160
6D_1M		0.0416	0.0560	0.0515	0.1061		0.0472	0.8552
6D_2M		0.0336	0.0360	0.0195	0.0464		0.0331	0.4896
6D_3M	0.0421	0.0608	0.0805	0.0787	0.1691		0.0459	0.7768
6D_1E	0.0389	0.0256	0.0296	0.0224	0.0411		0.0309	0.4659
6D_2E	0.0987	0.0432	0.0693	0.0541	0.1445		0.0472	0.5747
6D_3E	0.1283	0.0755	0.0493	0.0803	0.1379		0.0616	0.7205
7A_1M	0.0317	0.0576	0.0565	0.1419	0.1368	0.0309	0.1323	0.5864
7A_2M		0.0173	0.0235	0.0493	0.0416	0.0133	0.0440	0.2339
7A_3M		0.0120	0.0104	0.0221	0.0168		0.0291	0.1499
7A_1E	0.1149	0.0824	0.0880	0.2435	0.2869	0.0317	0.1803	0.6821
7A_2E	0.0424	0.0232	0.0971	0.2448	0.2037	0.0107	0.0547	0.2123
7A_3E	0.0387	0.0189	0.0397	0.0600	0.0904	0.0221	0.0712	0.2899

7B_1M		0.0429	0.1195	0.1019	0.2877		0.0685	1.0536
7B_2M		0.0352	0.1408	0.1440	0.2893		0.0643	1.2819
7B_3M	0.0389	0.1040	0.2059	0.1421	0.3173		0.1491	1.8712
7B_1E	0.0235	0.0301	0.0595	0.1101	0.1765		0.0493	0.9509
7B_2E	0.0675	0.0387	0.1200	0.0837	0.2757		0.0805	1.0813
7B_3E	0.1003	0.0648	0.1720	0.1373	0.2528		0.1376	1.6595
8A_2M		0.0104	0.0771	0.1555	0.0963	0.0091	0.0539	0.0859
8A_3M	0.0683	0.0587	0.2861	0.5019	0.4029	0.0221	0.2331	0.3416
8A_1E	0.0795	0.0488	0.2232	0.3904	0.4128	0.0147	0.1907	0.3648
8A_3E	0.0701	0.0424	0.2043	0.3827	0.3077	0.0139	0.2021	0.3448
8B_1M	0.0392	0.0139	0.0165	0.0315	0.0333		0.0435	0.1888
8B_2M	0.1251	0.0707	0.0261	0.0824	0.0829		0.1744	0.6645
8B_3M		0.0197	0.0104	0.0509	0.0437		0.0717	0.2733
8B_1E	0.1125	0.0667	0.0480	0.0307	0.0859		0.2472	1.0304
8B_2E	0.1275	0.0688	0.0595	0.1464	0.2128	0.0216	0.2309	0.6123
8B_3E	0.1037	0.0643	0.0440	0.0405	0.0899		0.2120	0.9437
10_1M	0.1085	0.0653	0.1459	0.2147	0.3181		0.2800	0.5389
10_2M	0.0760	0.0677	0.1251	0.1888	0.3099		0.2624	0.6192
10_3M	0.1229	0.0744	0.4392	0.2901	0.6653		0.3171	0.5704
10_1E	0.1144	0.0635	0.1264	0.2747	0.3181		0.2485	0.6581
10_2E	0.1888	0.0568	0.2405	0.5120	0.4627		0.3629	0.3328
10_3E	0.1621	0.0584	0.2195	0.4403	0.4611		0.2757	0.4784
11_4M	0.0376	0.1416	0.3605	0.2395	0.3853		0.4283	1.7219
11_2M		0.0173	0.0307	0.1080	0.0957	0.0181	0.0653	0.3675
11_3M		0.0152	0.0427	0.0459	0.1179		0.0413	0.3336
11_1E	0.0880	0.0685	0.0435	0.0285	0.0989		0.2005	1.2896
11_2E	0.0584	0.0328	0.1211	0.0835	0.2845	0.0128	0.1256	0.5120
11_3E		0.0685	0.0269	0.1155	0.1205	0.0325	0.1760	0.8011
13A_1M	0.0192	0.0232	0.0680	0.2477	0.1744		0.0867	0.2288
13A_2M	0.0608	0.0573	0.2680	0.4952	0.5851		0.3275	0.7213
13A_3M	0.0336	0.0693	0.2384	0.3317	0.4957		0.3269	0.7317
13A_1E	0.0949	0.0597	0.2539	0.5952	0.6379		0.2917	0.5792
13A_2E	0.1013	0.0531	0.2675	0.4160	0.6237		0.3304	0.7872
13A_3E	0.0701	0.0581	0.3280	0.4613	0.6400		0.3376	0.7675
13B_1M		0.0397	0.1523	0.0931	0.2272		0.1411	0.2957
13B_2M		0.0235	0.0627	0.0411	0.0701	0.0163	0.0680	0.2464
13B_3M	0.0920	0.0859	0.1840	0.0984	0.1544		0.2955	0.9443
13B_1E	0.1773	0.1173	0.3365	0.1603	0.2291		0.4283	1.1440
13B_2E	0.1912	0.1027	0.1059	0.0501	0.0752		0.3835	0.7629
13B_3E	0.0419	0.0392	0.0528	0.0355	0.0483	0.0291	0.1349	0.3816
14_1M		0.0651	0.3051	0.3176	0.6165	0.0144	0.0800	0.6173
14_2M	0.0203	0.0451	0.0507	0.0549	0.1664		0.0365	0.4347
14_3M		0.0123	0.0195	0.0389	0.0536		0.0197	0.1283
14_1E		0.0213	0.1000	0.0819	0.1501	0.0165	0.0312	0.3133
14_2E	0.0523	0.0725	0.0523	0.1235	0.1395	0.0139	0.0619	0.5960
14_3E	0.0408	0.0707	0.2472	0.3696	0.5005		0.0800	0.4941

15A_1M	0.1272	0.0787	0.1021	0.0480	0.0835		0.5192	1.1515
15A_2M		0.0243	0.0456	0.0483	0.1384		0.0896	0.3728
15A_3M	0.0488	0.0709	0.2221	0.3000	0.6448		0.2859	0.9923
15A_1E	0.0949	0.0627	0.0653	0.1936	0.2717		0.2528	0.6419
15A_2E	0.0779	0.0603	0.1877	0.4408	0.7179		0.2760	0.5720
15A_3E	0.0211	0.0149	0.0237	0.1067	0.0928		0.0797	0.2181
15B_1M	0.0723	0.1109	0.1720	0.1208	0.3093		0.1477	0.5208
15B_2M		0.0328	0.0656	0.0675	0.1893	0.0139	0.0467	0.2421
15B_3M		0.0336	0.0947	0.0859	0.2603		0.0781	0.3043
15B_1E	0.1325	0.1640	0.3085	0.2709	0.1691		0.2696	0.8491
15B_2E		0.0539	0.0909	0.0603	0.1496		0.1200	0.5744
15B_3E	0.0773	0.0635	0.0677	0.4544	0.2307		0.1760	0.3965
15C_1M	0.0685	0.0592	0.0539	0.1379	0.2139	0.0341	0.3208	0.3363
15C_2M	0.1896	0.1365	0.1141	0.0440	0.1003	0.0485	0.6661	0.5600
15C_3M		0.0123	0.0213	0.0301	0.0680	0.0173	0.0797	0.1205
15C_1E	0.1288	0.1107	0.0573	0.0491	0.0576	0.0336	0.5541	0.7248
15C_2E		0.0139	0.0141	0.0093	0.0261	0.0155	0.0952	0.1549
15C_3E	0.1317	0.1213	0.0669	0.0392	0.0389	0.0709	0.6107	0.7955
16_1M		0.0123	0.0144	0.0512	0.0749		0.0416	0.1539
16_2M		0.0987	0.0648	0.0560	0.1467	0.0472	0.1968	0.6904
16_3M		0.0429	0.0835	0.0632	0.1995	0.0133	0.0627	0.3547
16_1E		0.0293	0.0192	0.0240	0.0477	0.0280	0.0619	0.3416
16_2E		0.0147	0.0187	0.0437	0.0779	0.0091	0.0317	0.1269
16_3E	0.0344	0.0331	0.0440	0.1328	0.1459	0.0165	0.0461	0.2109
18_1M		0.0101	0.0077	0.0445	0.0408	0.0067	0.0821	0.1096
18_2M		0.0128	0.0101	0.0547	0.0491	0.0056	0.1043	0.1144
18_3M		0.0525	0.0683	0.1821	0.2928	0.0149	0.4456	0.6056
18_1E	0.0632	0.0595	0.0341	0.1253	0.1323	0.0328	0.4808	0.5037
18_2E		0.0301	0.0523	0.0475	0.0523	0.0235	0.2088	0.3299
18_3E	0.0539	0.0821	0.0432	0.0317	0.0896	0.0293	0.5136	0.6403
19_1M		0.0427	0.0787	0.0741	0.1075		0.1704	0.4443
19_2M	0.0661	0.0955	0.2232	0.1411	0.3893		0.3264	0.6853
19_3M		0.0405	0.0755	0.0685	0.1139		0.1136	0.3725
19_1E	0.0453	0.0744	0.1728	0.0995	0.4408	0.0528	0.2395	0.6349
19_2E	0.0539	0.0659	0.1595	0.1485	0.4307	0.0291	0.2408	0.5253
19_3E	0.0832	0.0648	0.0533	0.0739	0.1376	0.0485	0.2072	0.5696

ID	Creatine	Creatine phosphate	Creatinine	Dimethylamine	Formate	Fucose	Fumarate	Galactose
4A_1M	0.0552	0.0213	0.0333	0.0016	0.0336	0.4221		0.8424
4A_2M	0.0603	0.0312	0.0363	0.0021	0.0405	0.4147	0.0040	0.9120
4A_3M	0.0253	0.0115	0.0184	0.0019	0.0229	0.2237	0.0032	0.3528
4A_1E	0.0112	0.0053	0.0077	0.0016	0.0141	0.0507		0.1416
4A_2E	0.0376	0.0112	0.0203	0.0005	0.0341	0.2397		0.6112
4A_3E	0.0069	0.0080	0.0216	0.0008	0.0163	0.1200	0.0021	0.2848
4B_1M	0.0523	0.0051	0.0323	0.0016	0.0235	0.2883	0.0027	0.8944
4B_2M	0.0669	0.0173	0.0427		0.0336	0.3984		0.9091
4B_3M	0.0245	0.0024	0.0136	0.0011	0.0120	0.1147		0.3216

4B_1E	0.0104		0.0104		0.0205	0.0568		0.1624
4B_2E	0.0408	0.0061	0.0237	0.0008	0.0181	0.1563	0.0053	0.5869
4B_3E	0.0099		0.0067		0.0115	0.0296		0.0944
5_1M	0.0336		0.0179		0.0181	0.1379	0.0037	0.3208
5_2M	0.0288		0.0163	0.0011	0.0168	0.1427	0.0024	0.3165
5_3M	0.0307		0.0187	0.0019	0.0155	0.1384	0.0021	0.3773
5_1E	0.0160		0.0093		0.0096	0.0507	0.0024	0.2432
5_2E	0.0576		0.0299	0.0013	0.0400	0.2317	0.0037	0.7616
5_3E	0.0339		0.0213	0.0011	0.0205	0.0912	0.0037	0.4979
6A_1M	0.0125	0.0024	0.0083	0.0008	0.0117	0.1056	0.0024	0.3061
6A_2M	0.0448	0.0149	0.0219	0.0024	0.0355	0.3099	0.0043	0.7997
6A_3M	0.0280	0.0117	0.0149		0.0256	0.1576		0.4747
6A_1E	0.0851	0.0208	0.0395	0.0021	0.0552	0.4248	0.0096	1.5341
6A_2E	0.1019	0.0381	0.0515	0.0037	0.0549	0.5288	0.0104	1.3816
6A_3E	0.0389	0.0104	0.0133		0.0347	0.2197		0.6248
6B_1M	0.0496	0.0192	0.0301	0.0016	0.0387	0.3675		0.9216
6B_2M	0.0509	0.0192	0.0312	0.0021	0.0381	0.5765	0.0045	1.3683
6B_3M	0.0549	0.0133	0.0285	0.0016	0.0413	0.4251		0.7856
6B_1E	0.0157	0.0029	0.0072	0.0011	0.0160	0.1019	0.0032	0.2621
6B_2E	0.0536	0.0131	0.0264		0.0373	0.3344	0.0069	0.9853
6B_3E	0.0835	0.0344	0.0547	0.0029	0.0421	0.5043	0.0080	1.2019
6C_4M	0.0232	0.0035	0.0077	0.0013	0.0168	0.0928		0.2512
6C_2M	0.0912	0.0157	0.0509		0.0376	0.2557	0.0053	1.0987
6C_3M	0.0317		0.0165		0.0144	0.1429		0.3240
6C_1E	0.0488		0.0211	0.0032	0.0259	0.1811	0.0037	0.9440
6C_2E	0.0299	0.0029	0.0152		0.0149	0.1464	0.0021	0.3787
6C_3E	0.0907	0.0043	0.0405		0.0371	0.3709	0.0037	1.1408
6D_1M	0.0352		0.0291		0.0192	0.1400		0.5091
6D_2M	0.0291		0.0221		0.0283	0.1008		0.4467
6D_3M	0.0480		0.0355	0.0019	0.0320	0.2483		0.7763
6D_1E	0.0205		0.0160		0.0213	0.0696		0.2573
6D_2E	0.0376		0.0280		0.0253	0.1173		0.4355
6D_3E	0.0525	0.0291	0.0328		0.0317	0.1488	0.0037	0.7224
7A_1M	0.0467		0.0208	0.0013	0.0256	0.4475		0.8531
7A_2M	0.0144		0.0069		0.0123	0.1053		0.2701
7A_3M	0.0088		0.0051		0.0101	0.0781	0.0021	0.1643
7A_1E	0.0512		0.0248		0.0203	0.3995	0.0043	0.8176
7A_2E	0.0205		0.0109		0.0131	0.1363	2.6667	0.3176
7A_3E	0.0149		0.0075		0.0109	0.1309	0.0019	0.3552
7B_1M	0.0229		0.0125	0.0016	0.0176	0.1685	2.6667	0.3611
7B_2M	0.0245		0.0139		0.0248	0.2011	0.0019	0.5843
7B_3M	0.0504		0.0312	0.0029	0.0355	0.3696	0.0040	1.1299
7B_1E	0.0171		0.0104		0.0133	0.1133	0.0037	0.3419
7B_2E	0.0237		0.0163	0.0019	0.0216	0.1819	0.0029	0.5357
7B_3E	0.0475		0.0301	0.0128	0.0275	0.2600	0.0035	0.8141
8A_2M	0.0072		0.0040	0.0008	0.0056	0.0619	0.0021	0.2027

8A_3M	0.0288		0.0168		0.0149	0.2509	0.0072	0.8144
8A_1E	0.0277		0.0171		0.0165	0.2835	0.0064	0.6835
8A_3E	0.0299		0.0176		0.0155	0.2605	0.0093	0.7597
8B_1M	0.0091		0.0115		0.0136			0.1747
8B_2M	0.0219		0.0451	0.0013	0.0149	0.0288	0.0043	0.7608
8B_3M	0.0115		0.0163	2.6667	0.0120	0.0093		0.2645
8B_1E	0.0299		0.0456	0.0016	0.0165	0.0219	0.0032	0.7051
8B_2E	0.0261		0.0421	0.0013	0.0149	0.0360	0.0037	0.5891
8B_3E	0.0272		0.0488	0.0011	0.0165	0.0264		0.7165
10_1M	0.0240		0.0368	0.0011	0.0259	0.0379	0.0019	0.5955
10_2M	0.0232		0.0472	0.0016	0.0200	0.0629	0.0029	0.8280
10_3M	0.0373		0.0443	0.0024	0.0227			0.8760
10_1E	0.0229		0.0437		0.0227	0.0659		0.5736
10_2E	0.0243		0.0427		0.0227			0.6776
10_3E	0.0203		0.0445	0.0016	0.0192	0.0528	0.0048	0.7411
11_4M	0.0608		0.0475		0.0277	0.1061	0.0064	1.4883
11_2M	0.0136		0.0088	0.0011	0.0093			0.2403
11_3M	0.0117		0.0077		0.0139		0.0024	0.1915
11_1E	0.0624		0.0400		0.0147	0.0304	0.0029	0.7421
11_2E	0.0216		0.0187	0.0008	0.0155		0.0024	0.4531
11_3E	0.0464		0.0320	0.0011	0.0149	0.0499	0.0035	0.7445
13A_1M	0.0072		0.0123	0.0008	0.0165	0.1408		0.2637
13A_2M	0.0139		0.0296	0.0013	0.0309	0.3272		0.7091
13A_3M	0.0144		0.0309		0.0275	0.3568	0.0032	0.7877
13A_1E	0.0253		0.0317	0.0013	0.0312	0.3675	0.0053	0.7680
13A_2E	0.0267		0.0317		0.0365	0.3283	0.0059	0.7421
13A_3E	0.0192		0.0304	0.0011	0.0304	0.2872	0.0045	0.6037
13B_1M	0.0069	0.0027	0.0144		0.0205	0.1171		0.2629
13B_2M	0.0064		0.0120	0.0011	0.0131	0.0549		0.1464
13B_3M	0.0200		0.0445	0.0027	0.0256	0.3104	0.0032	0.5693
13B_1E	0.0296		0.0581		0.0443	0.3192	0.0088	0.5227
13B_2E	0.0288		0.0557	0.0032	0.0339	0.2509		0.8024
13B_3E	0.0168		0.0211		0.0171	0.1181		0.2360
14_1M	0.0376	0.0237	0.0472	0.0037	0.0109	0.6389	0.0032	0.2509
14_2M	0.0291	0.0141	0.0269	0.0019	0.0147	0.3784		0.4400
14_3M	0.0069	0.0043	0.0069		0.0085	0.1160		0.1325
14_1E	0.0101	0.0083	0.0115		0.0109	0.1536		0.2261
14_2E	0.0315	0.0285	0.0355	0.0011	0.0112	0.5859	0.0048	0.2256
14_3E	0.0368	0.0229	0.0325	0.0019	0.0171	0.4851	0.0043	0.1677
15A_1M	0.0163		0.0379	0.0024	0.0397	0.3493	0.0040	1.0131
15A_2M	0.0083		0.0133		0.0200	0.1277	0.0032	0.2627
15A_3M	0.0261		0.0360		0.0325	0.3096	0.0043	0.9403
15A_1E	0.0189		0.0387		0.0421	0.2827	0.0080	1.0117
15A_2E	0.0251		0.0344		0.0328	0.3416	0.0069	0.7352
15A_3E	0.0075		0.0112		0.0133	0.0669	0.0027	0.1619
15B_1M	0.0195		0.0387	0.0037	0.0419	0.3763	0.0027	0.9131

15B_2M	0.0077		0.0176		0.0189	0.1349		0.2323
15B_3M	0.0064		0.0200	0.0011	0.0216	0.1277	0.0016	0.3179
15B_1E	0.0389		0.0611	0.0037	0.0677	0.6997	0.0061	1.4704
15B_2E	0.0176		0.0355		0.0152	0.2325	0.0048	0.5109
15B_3E	0.0165		0.0339		0.0283	0.2469	0.0032	0.4200
15C_1M	0.0392		0.0395		0.0221	0.9067	0.0048	0.3848
15C_2M	0.0973		0.0675		0.0445	2.2643	0.0152	1.7173
15C_3M	0.0085		0.0080		0.0091	0.1624	0.0016	0.2365
15C_1E	0.0704		0.0533		0.0331	1.5901	0.0123	1.5677
15C_2E	0.0139		0.0107		0.0104	0.2608	0.0021	0.2819
15C_3E	0.0872		0.0627	0.0027	0.0357	1.9173	0.0093	1.2701
16_1M	0.0083		0.0104		0.0189	0.2664		0.1285
16_2M	0.0360		0.0448	0.0029	0.0627	1.4053	0.0037	0.9296
16_3M	0.0179		0.0245	0.0019	0.0357	0.4931	0.0024	0.3472
16_1E	0.0144		0.0181		0.0296	0.5149	0.0021	0.2411
16_2E	0.0075	0.0021	0.0099		0.0163	0.2267		0.1448
16_3E	0.0149		0.0205		0.0373	0.4795	0.0024	0.2837
18_1M	0.0035	0.0016	0.0069		0.0136	0.1141	0.0013	0.0899
18_2M	0.0040		0.0075		0.0139	0.1165		0.1261
18_3M	0.0213		0.0328		0.0475	0.7005	0.0045	0.5611
18_1E	0.0152		0.0323		0.0427	0.5853	0.0027	0.6851
18_2E	0.0093		0.0168		0.0280	0.2368	0.0032	0.3059
18_3E	0.0205		0.0464		0.0464	0.8400	0.0043	0.7819
19_1M	0.0184	0.0035	0.0205		0.0272	0.1984	0.0048	0.4731
19_2M	0.0264		0.0347	0.0035	0.0429	0.5523	0.0035	0.8168
19_3M	0.0120		0.0155	0.0019	0.0245	0.1611		0.3131
19_1E	0.0285		0.0339	0.0024	0.0344	0.5317	0.0040	0.8141
19_2E	0.0251	0.0072	0.0315		0.0395	0.2984	0.0048	0.4507
19_3E	0.0237	0.0051	0.0296		0.1075	0.3304	0.0043	0.6061

ID	Gluconate	Glucose	Glutamate	Glutamine	Isoleucine	Lactate	Lactose	Leucine
4A_1M	0.6872	1.6317	1.2600	0.3291	0.0107	0.0619	167.8688	0.0355
4A_2M	0.5960	1.5419	1.3328	0.3773	0.0155	0.0461	187.0475	0.0317
4A_3M	0.3576	0.4339	0.5421	0.1141	0.0064	0.0499	73.2669	0.0152
4A_1E	0.1104	0.3013	0.2643	0.0648		0.0251	29.0309	0.0091
4A_2E	0.4336	0.8435	0.7259	0.2715	0.0064	0.0501	96.8515	0.0115
4A_3E	0.2675	0.5128	0.5107	0.1339	0.0067	0.0464	56.8864	0.0171
4B_1M	0.5501	0.9781	1.2248	0.3869		0.0589	176.4208	0.0117
4B_2M	0.6040	2.2827	1.2795	0.5192		0.0675	197.7440	0.0115
4B_3M	0.2307	0.4107	0.5499	0.2211		0.0291	73.2712	
4B_1E	0.1403	0.6421	0.4365	0.1128		0.0157	42.2368	
4B_2E	0.4275	1.3013	0.8632	0.2792		0.1467	139.0163	0.0117
4B_3E	0.1184	0.2453	0.2677	0.0605		0.0259	26.4107	0.0037
5_1M	0.3315	0.3920	0.6160	0.2312		0.0453	91.8197	0.0064
5_2M	0.2045	0.5989	0.6304	0.2384		0.0501	83.8109	
5_3M	0.2925	0.5584	0.6312	0.2667		0.0373	93.7608	0.0147
5_1E	0.1861	0.2493	0.2987	0.0885		0.0349	41.3701	

5_2E	0.4901	0.8376	0.9531	0.2555		0.0661	154.5664	0.0131
5_3E	0.2989	0.8136	0.6475	0.1507		0.0499	81.6195	0.0088
6A_1M	0.1835	0.1773	0.2683	0.0643		0.0768	39.9563	0.0075
6A_2M	0.5584	1.2176	0.8688	0.2427	0.0075	0.1021	128.9341	0.0173
6A_3M	0.2821		0.4331	0.1520		0.0296	69.4851	0.0120
6A_1E	0.9843	2.2269	2.1421	0.4611	0.0267	0.1608	211.1035	0.0243
6A_2E	0.9368	2.6669	2.0947	0.4896	0.0304	0.1765	245.6088	0.0608
6A_3E	0.5568		0.8677	0.2568	0.0085	0.0573	101.4883	0.0165
6B_1M	0.5304	1.0787	0.9760	0.3709	0.0083	0.0587	153.1869	0.0232
6B_2M	0.7760	2.0731	1.6067	0.6235	0.0117	0.0912	199.8413	0.0331
6B_3M	0.6227	1.2299	1.2304	0.4923	0.0203	0.0552	149.1344	0.0387
6B_1E	0.2176	0.5176	0.3669	0.1283	0.0064	0.0307	52.7600	0.0072
6B_2E	0.5771	3.1768	1.2907	0.3851	0.0168	0.1235	153.9960	0.0235
6B_3E	0.5851	4.1675	1.7341	0.5525	0.0253	0.1435	215.3563	0.0333
6C_4M	0.1760	0.2424	0.4579	0.1344		0.0309	60.2760	0.0061
6C_2M	0.3824	1.2197	1.3547	0.2181	0.0117	0.0584	144.8304	0.0149
6C_3M	0.1813	0.5125	0.4195	0.1331		0.0299	75.6104	0.0064
6C_1E	0.3419	1.0400	0.8523	0.2021	0.0069	0.1048	128.3352	0.0099
6C_2E	0.1973	0.9021	0.6379	0.1776		0.0317	72.9771	0.0069
6C_3E	0.6280	1.3976	1.5075	0.2128	0.0080	0.0845	201.1669	0.0096
6D_1M	0.4915	0.9365	0.6933	0.6104	0.0053	0.0333	100.8053	0.0136
6D_2M	0.2717	0.5117	0.6141	0.4971		0.0229	87.9947	0.0080
6D_3M	0.4467	1.0803	1.1309	0.7536	0.0069	0.0560	160.5717	0.0168
6D_1E	0.1760	0.6219	0.4672	0.3253	0.0037	0.0195	63.6483	0.0067
6D_2E	0.3176	0.9768	0.8131	0.3891	0.0069	0.0277	102.9936	0.0131
6D_3E	0.3824	2.3400	1.3248	0.7371	0.0093	0.0429	167.7507	0.0285
7A_1M	0.2867	1.0576	0.9909	0.2432	0.0048	0.0408	150.4008	0.0141
7A_2M	0.1184	0.3048	0.3251	0.1064		0.0184	42.6619	0.0072
7A_3M	0.0445	0.2648	0.2173	0.0557		0.0080	26.7195	0.0032
7A_1E	0.2616	1.8496	1.3037	0.3357	0.0104	0.0757	197.9160	0.0211
7A_2E	0.1381	0.5395	0.4333	0.1091	0.0048	0.0328	57.2603	0.0077
7A_3E	0.1144	0.4859	0.4872	0.1272		0.0227	62.3640	0.0101
7B_1M	0.2608	0.7485	0.5229	0.4333		0.0221	97.7035	0.0173
7B_2M	0.3032	0.6339	0.5288	0.3525		0.0259	104.7264	0.0152
7B_3M	0.5229	1.2981	0.9048	0.7419		0.0395	181.8691	0.0181
7B_1E	0.2139	0.7451	0.4589	0.2923		0.0237	74.5528	0.0051
7B_2E	0.2803	0.8461	0.7515	0.4755	0.0053	0.0437	102.2733	0.0117
7B_3E	0.4051	1.7501	1.1763	0.8453		0.0624	185.9453	0.0088
8A_2M	0.0291	0.1805	0.1600	0.0309		0.0589	31.7408	
8A_3M	0.0840	0.8293	0.7283	0.1443	0.0069	0.1165	138.4000	0.0112
8A_1E	0.1581	0.8635	0.7096	0.1269	0.0067	0.1320	135.3315	0.0093
8A_3E	0.1408	0.8221	0.6464	0.0997	0.0043	0.1675	127.4744	0.0085
8B_1M	0.0333	0.3640	0.4451	0.1651	0.0048	0.0125	42.1691	0.0080
8B_2M	0.1171	1.3251	1.7843	0.7691	0.0069	0.0445	174.0733	0.0269
8B_3M	0.0624	0.4296	0.4819	0.1765		0.0165	62.0757	0.0085
8B_1E	0.0528	1.2933	1.8472	0.7440	0.0061	0.0704	184.1635	0.0195

8B_2E	0.0829	1.6811	1.9091	0.5677	0.0125	0.0499	200.0443	0.0371
8B_3E	0.0624	0.8781	1.7408	0.5245	0.0109	0.0509	163.3992	0.0331
10_1M	0.1224	1.2240	1.9973	0.6864	0.0173	0.0349	177.6560	0.0469
10_2M	0.1661	1.7227	2.0856	1.0872	0.0133	0.0579	190.9381	0.0381
10_3M	0.0816	1.6312	2.1131	0.8640	0.0131	0.0717	154.2947	0.0421
10_1E	0.1616	1.4323	1.5997	0.8805	0.0059	0.0760	172.3885	0.0205
10_2E	0.0731	1.5731	1.6573	0.5077	0.0056	0.0392	168.1941	0.0160
10_3E	0.1251	1.5835	1.6131	0.5413	0.0061	0.0611	167.2293	0.0237
11_4M	0.2781	2.0227	1.8683	0.6907	0.0035	0.0704	295.3853	0.0336
11_2M	0.1053	0.1392	0.3376	0.1067		0.0248	61.3277	0.0075
11_3M	0.0315	0.2720	0.2272	0.0656		0.0216	42.9827	
11_1E	0.0629	0.7760	1.0165	0.2253	0.0115	0.0901	195.2333	0.0328
11_2E	0.0749	0.6891	0.6259	0.1331	0.0075	0.0451	100.9176	0.0080
11_3E	0.1461	0.7715	0.7843	0.2125		0.1437	170.3680	0.0096
13A_1M	0.2333	0.3280	0.4688	0.2213	0.0051	0.0181	52.6376	0.0099
13A_2M	0.4584	1.2792	1.3829	0.8443	0.0061	0.0485	162.7005	0.0211
13A_3M	0.5181	1.0888	1.2909	0.7688	0.0051	0.0501	164.8813	0.0219
13A_1E	0.6424	1.6515	1.4405	0.5395	0.0099	0.0781	174.1184	0.0304
13A_2E	0.5949	1.3280	1.2805	0.3656	0.0085	0.0939	156.3493	0.0259
13A_3E	0.5067	1.5728	1.3813	0.5480	0.0072	0.0664	169.3283	0.0197
13B_1M	0.2072	0.4237	0.6613	0.2675		0.0323	98.2205	0.0045
13B_2M	0.1325	0.3747	0.3885	0.1779		0.0152	47.5939	
13B_3M	0.3645	1.2141	1.3323	0.6592	0.0069	0.0771	198.4203	0.0120
13B_1E	0.5125	1.9480	2.7896	0.8739	0.0107	0.0728	246.2469	0.0096
13B_2E	0.4147	1.8179	1.8885	0.6779	0.0093	0.0736	234.6984	0.0117
13B_3E	0.2267	0.6819	0.7421	0.1995		0.0267	90.5384	0.0048
14_1M	0.8968	0.9576	1.3056	0.4651	0.0093	0.0707	156.9237	0.0309
14_2M	0.5621	0.6813	0.7680	0.4091		0.0461	107.2867	0.0184
14_3M	0.1608	0.1928	0.2339	0.0901		0.0163	28.0520	0.0051
14_1E	0.2299	0.6491	0.4325	0.1296	0.0035	0.0213	49.7896	0.0085
14_2E	0.7579	1.6315	1.1987	0.3701	0.0037	0.0811	165.1320	0.0144
14_3E	0.7181	1.6171	1.1531	0.3061	0.0069	0.0773	161.1976	0.0216
15A_1M	0.8253	1.5755	2.2877	1.1483	0.0123	0.0965	219.9576	0.0320
15A_2M	0.2024	0.4384	0.5787	0.2363		0.0205	57.5424	0.0043
15A_3M	0.5773	0.9899	1.6405	0.4600	0.0104	0.0925	172.9949	0.0216
15A_1E	0.7357	1.5269	1.3821	0.5032	0.0107	0.1032	160.6107	0.0243
15A_2E	0.6459	1.5275	1.4280	0.2997	0.0088	0.1456	160.1512	0.0171
15A_3E	0.1205	0.3157	0.3963	0.1011		0.0448	43.7965	0.0056
15B_1M	0.5659	1.2117	1.5741	0.4304	0.0096	0.0693	209.5560	0.0131
15B_2M	0.1816	0.3211	0.5197	0.1888		0.0304	74.3072	
15B_3M	0.2115	0.4621	0.6616	0.1829		0.0605	84.2696	0.0037
15B_1E	0.6960	1.8517	2.1611	0.6296		0.1205	305.9555	
15B_2E	0.3872	0.9011	1.1557	0.2885	0.0093	0.0672	130.5499	0.0083
15B_3E	0.4667	1.1021	0.9587	0.2277	0.0083	0.0933	141.6344	0.0171
15C_1M	0.5963	0.1624	0.8184	0.2267	0.0053	0.1064	156.4368	0.0163
15C_2M	1.0133	1.0837	2.0765	0.5909	0.0109	0.2152	316.9643	0.0216

15C_3M	0.1179	0.0435	0.2915	0.0805		0.0245	34.6827	0.0040
15C_1E	1.0043	0.2776	1.8563	0.3899		0.3573	243.5264	0.0096
15C_2E	0.1475	0.0859	0.3264	0.0672		0.0291	43.9381	
15C_3E	1.1048	0.7043	2.0781	0.5200	0.0117	0.1963	255.9277	0.0139
16_1M	0.1165	0.0541	0.0523			0.0149	32.6552	
16_2M	0.7843	0.3811	0.6435	0.1099		0.0485	210.2747	0.0067
16_3M	0.3451	0.3197	0.5227	0.1763	0.0048	0.0461	90.2083	0.0077
16_1E	0.2803	0.3904	0.4613	0.0891		0.0285	75.9997	0.0037
16_2E	0.1512	0.1200	0.1200			0.0208	34.7544	0.0040
16_3E	0.3336	0.2496	0.5813	0.1323		0.0552	77.8107	0.0123
18_1M	0.1059	0.0963	0.1147	0.0352		0.0200	28.0947	0.0059
18_2M	0.1256	0.0944	0.1128	0.0227		0.0256	31.2104	
18_3M	0.8507	0.4440	0.5003	0.1011	0.0115	0.1805	137.1973	0.0163
18_1E	0.5805	0.7896	0.8099	0.2792	0.0085	0.1155	145.6579	0.0155
18_2E	0.2227	0.5624	0.4616	0.1531		0.0576	79.3173	
18_3E	0.7237	0.8515	0.6515	0.2285	0.0125	0.1043	187.9211	0.0189
19_1M	0.1613	0.4200	0.7411	0.1512	0.0067	0.0781	99.4573	0.0072
19_2M	0.3816	0.5544	0.9784	0.2939	0.0075	0.1445	202.0507	0.0176
19_3M	0.1213	0.2405	0.4312	0.1373		0.0315	79.7515	
19_1E	0.3147	0.7571	1.1211	0.1824	0.0083	0.1053	197.0381	0.0080
19_2E	0.2288	0.9048	0.8973	0.1384	0.0051	0.1173	172.0645	0.0181
19_3E	0.2987	0.6728	0.9675	0.1488	0.0069	0.0405	161.8037	0.0176

ID	Malonate	Methanol	Methionine	N-Acetylglucosamine	O-Phosphocholine	Pantothenate	Pyruvate	Succinate
4A_1M	0.0504	0.6451	0.0184	1.1235	0.3176	0.0099		0.0120
4A_2M	0.0712	0.9205	0.0184	1.1045	0.3992	0.0117		0.0136
4A_3M	0.0272	4.6429	0.0112	0.5019	0.1576	0.0051		0.0045
4A_1E	0.0149	71.5755	0.0045	0.1712	0.0600	0.0029		2.6667
4A_2E	0.0333	1.3488	0.0059	0.5608	0.2099	0.0104		0.0069
4A_3E	0.0179	1.1357	0.0053	0.3576	0.1264	0.0053		0.0027
4B_1M	0.0467	1.2947	0.0115	0.6715	0.2224	0.0144		0.0179
4B_2M	0.0707	1.5277	0.0059	0.6437	0.2973	0.0181		0.0232
4B_3M	0.0195	1.5331	0.0053	0.2619	0.0576	0.0088		0.0080
4B_1E	0.0099	62.3931	0.0027	0.1787	0.0851			0.0032
4B_2E	0.0339	1.1544	0.0099	0.5440	0.2395	0.0155	0.0141	0.0075
4B_3E	0.0085	1.0264	0.0027	0.1048	0.0453		0.0021	0.0021
5_1M	0.0208	0.3888	0.0093	0.3123	0.0507	0.0109		
5_2M	0.0219	2.2664	0.0075	0.2909	0.0651	0.0104		0.0072
5_3M	0.0245	2.7123	0.0067	0.3539	0.0221	0.0093	0.0032	0.0053
5_1E	0.0123	0.9925	0.0027	0.1659	0.0387	0.0056		0.0040
5_2E	0.0235	0.9245	0.0144	0.5805	0.0731	0.0189		0.0259
5_3E	0.0147	2.6005	0.0083	0.3949	0.0592	0.0099		0.0077
6A_1M	0.0176	115.9291	0.0027	0.2299	0.0493		0.0032	0.0019
6A_2M	0.0443	532.2523	0.0117	0.7405	0.1840	0.0085	0.0117	0.0088
6A_3M		229.6795		0.4101	0.1341			0.0043
6A_1E	0.0995	102.8691	0.0315	1.6328	0.3979		0.0216	0.0144
6A_2E	0.0712	7.2701	0.0219	1.6536	0.5013	0.0187	0.0061	0.0123

6A_3E	0.0467	362.3619	0.0149	0.7696	0.1824	0.0120		0.0067
6B_1M	0.0357	5.5579	0.0195	0.8123	0.3051	0.0099		0.0072
6B_2M	0.0459	4.2563	0.0131	1.0069	0.3736	0.0131	0.0080	0.0109
6B_3M	0.0616	14.2333	0.0088	0.8627	0.2405	0.0147		0.0088
6B_1E	0.0125	2.7328	0.0064	0.2845	0.0888	0.0037		0.0027
6B_2E	0.0352	466.7619	0.0109	0.9549	0.2851			0.0091
6B_3E	0.0509	8.6552	0.0267	1.1304	0.3981	0.0173	0.0144	0.0131
6C_4M	0.0141	3.0968	0.0080	0.1992	0.0187	0.0075		0.0051
6C_2M	0.0363	6.5331		0.4645	0.2253	0.0171		0.0149
6C_3M	0.0224	3.7525	0.0064	0.2043	0.0725	0.0056	0.0043	0.0061
6C_1E	0.0328	3.8267	0.0107	0.3760	0.1315	0.0133	0.0131	0.0120
6C_2E	0.0192	2.1589	0.0104	0.1939	0.1029	0.0091		0.0072
6C_3E	0.0496	19.4867	0.0088	0.5944	0.1659	0.0221	0.0168	0.0376
6D_1M	0.0131	3.8992	0.0157	0.5933	0.2349	0.0061		0.0069
6D_2M	0.0112	4.6752	0.0088	0.5416	0.2024			0.0029
6D_3M	0.0144	3.5656	0.0112	0.6909	0.3757	0.0088		0.0101
6D_1E	0.0085	59.3480	0.0093	0.2933	0.1301			0.0037
6D_2E	0.0091	6.1451	0.0075	0.6088	0.2229	0.0077		0.0056
6D_3E	0.0216	3.3547	0.0080	0.8579	0.4131	0.0067		0.0101
7A_1M	0.0803	1.6763	0.0155	0.4723	0.0632	0.0147		0.0099
7A_2M	0.0213	1.7893	0.0061	0.1587	0.0208	0.0056		0.0029
7A_3M	0.0144	1.0459	0.0016	0.1048	0.0211	0.0029		0.0019
7A_1E	0.0771	4.9464	0.0184	0.4728	0.1213	0.0173	0.0075	0.0120
7A_2E	0.0208	0.3117	0.0043	0.2245	0.0320	0.0077		0.0045
7A_3E	0.0219	1.4827	0.0077	0.2280	0.0376	0.0059		0.0032
7B_1M	0.0064	4.2792	0.0053	0.3245	0.0941	0.0045		0.0024
7B_2M	0.0072	4.6344	0.0163	0.3581	0.1035	0.0043		0.0027
7B_3M	0.0149	3.9293	0.0211	0.6269	0.1688	0.0051		0.0091
7B_1E	0.0077	2.4808	0.0048	0.2525	0.0744		0.0061	0.0027
7B_2E	0.0069	1.5333	0.0056	0.3360	0.1013	0.0037	0.0093	0.0035
7B_3E	0.0136	3.9128	0.0088	0.5803	0.1733	0.0056	0.0093	0.0067
8A_2M	0.0051	0.6227	0.0032	0.0576	0.0029		0.0045	0.0024
8A_3M	0.0264	0.3000	0.0091	0.2595	0.0227	0.0091	0.0136	0.0115
8A_1E	0.0387	0.3195	0.0131	0.2395	0.0440	0.0115	0.0099	0.0131
8A_3E	0.0208	0.1477	0.0083	0.2240	0.0251	0.0088	0.0123	0.0141
8B_1M	0.0232	1.1440	0.0043	0.1699	0.0573	0.0040		0.0027
8B_2M	0.0848	4.2869	0.0227	0.6565	0.2472	0.0072		0.0133
8B_3M	0.0344	4.3357	0.0048	0.3784	0.0453	0.0059		0.0051
8B_1E	0.0835	7.3749	0.0080	0.8853	0.2424	0.0131	0.0133	0.0133
8B_2E	0.0653	5.3979	0.0149	0.7115	0.2653	0.0133		0.0211
8B_3E	0.0619	3.0235		0.7344	0.2395	0.0120		0.0115
10_1M	0.0325	0.5232	0.0059	0.6109	0.2304	0.0109		0.0283
10_2M	0.0461	0.5869	0.0088	0.8400	0.3064	0.0091		0.0336
10_3M	0.0323	2.2011		0.9325	0.2624	0.0101		0.0259
10_1E	0.0243	2.7003	0.0139	0.5923	0.1907	0.0109		0.0248
10_2E	0.0216	0.4688	0.0179	0.8195	0.1971	0.0101		0.0123

10_3E	0.0168	0.3787	0.0200	0.7107	0.2613	0.0117		0.0261
11_4M	0.0685	1.3435	0.0061	1.2437	0.0229	0.0267		0.0395
11_2M	0.0141	2.7752	0.0056	0.2387	0.0053	0.0059		0.0035
11_3M	0.0101	2.6235	0.0056	0.2179	0.0056			0.0032
11_1E	0.0552	8.5259	0.0251	1.0381	0.0128	0.0184	0.0139	0.0099
11_2E	0.0181	5.1936	0.0123	0.3664	0.0064	0.0083	0.0075	0.0045
11_3E	0.0440	47.1248	0.0131	0.8784	0.0133	0.0160	0.0059	0.0237
13A_1M	0.0040	1.1792	0.0101	0.2093	0.0429	2.6667		0.0056
13A_2M	0.0123	0.3261	0.0163	0.4909	0.0728	0.0061		0.0387
13A_3M	0.0152	0.5328	0.0093	0.4859	0.0715	0.0120		0.0336
13A_1E	0.0171	1.6752	0.0080	0.5715	0.1043	0.0136	0.0099	0.0347
13A_2E	0.0141	1.4923	0.0091	0.6640	0.0973	0.0123	0.0112	0.0221
13A_3E	0.0117	0.2064	0.0096	0.5139	0.0733	0.0075	0.0091	0.0357
13B_1M	0.0149	5.2709	0.0075	0.4816	0.1051	0.0067		0.0035
13B_2M	0.0069	2.1931	0.0096	0.2429	0.0965			0.0024
13B_3M	0.0477	11.8616	0.0256	1.1299	0.1832	0.0107		0.0104
13B_1E	0.0491	0.5544		1.3693	0.3589	0.0189		0.0341
13B_2E	0.0485	9.9400	0.0261	1.2104	0.2331	0.0163		0.0109
13B_3E	0.0157	2.1451	0.0112	0.5237	0.0699	0.0061		0.0056
14_1M	0.0331	2.4621	0.0235	0.5848	0.5355	0.0659	0.0171	0.0123
14_2M	0.0480	0.4096	0.0117	0.4280	0.3931	0.0509		0.0107
14_3M	0.0128	0.8003	0.0024	0.1088	0.0856	0.0128		0.0011
14_1E	0.0085	30.1856	0.0056	0.1664	0.1949	0.0152	0.0035	0.0021
14_2E	0.0531	4.3325	0.0141	0.5379	0.5635	0.0368	0.0184	0.0083
14_3E	0.0280	2.3755	0.0144	0.5261	0.5813	0.0587	0.0096	0.0072
15A_1M	0.0432	4.3091	0.0152	0.9067	0.0480	0.0245		0.0440
15A_2M	0.0059	2.2640	0.0053	0.2200	0.0432	0.0043		0.0048
15A_3M	0.0248	4.6136	0.0232	0.6571	0.0925	0.0120		0.0277
15A_1E	0.0221	0.5371	0.0131	0.6656	0.0944	0.0093		0.0480
15A_2E	0.0235	3.0675	0.0120	0.6541	0.0933	0.0123		0.0448
15A_3E	0.0099	3.8248	0.0032	0.1741	0.0213	0.0027	0.0043	0.0045
15B_1M	0.0328	8.5117	0.0245	1.0976	0.3789	0.0136	0.0139	0.0101
15B_2M	0.0080	4.0883	0.0067	0.3509	0.1720	0.0040		0.0032
15B_3M	0.0085	4.2739	0.0069	0.4603	0.1648		0.0080	0.0035
15B_1E	0.0451	12.9355	0.0227	1.8781	0.6400	0.0235		0.0253
15B_2E	0.0176	7.9955	0.0235	0.6131	0.2779	0.0133		0.0104
15B_3E	0.0133	0.1251	0.0136	0.7267	0.2259	0.0085		0.0197
15C_1M	0.0339	4.5523	0.0075	0.4507	0.2576	0.0107	0.0160	0.0221
15C_2M	0.0733	79.7931	0.0203	1.2944	0.3507	0.0259	0.0427	0.0555
15C_3M	0.0067	3.4253	0.0061	0.1515	0.0320			0.0051
15C_1E	0.0597	13.3053	0.0155	1.1776	0.5016	0.0171	0.0451	0.0352
15C_2E	0.0104	7.1907	0.0027	0.1512	0.0325		0.0056	0.0083
15C_3E	0.0573	4.5643	0.0155	1.0928	0.2203	0.0211	0.0363	0.0437
16_1M	0.0107	2.9208	0.0064	0.1104	0.0283	0.0040	0.0021	0.0027
16_2M	0.0544	2.3747	0.0139	0.6757	0.4160	0.0136		0.0256
16_3M	0.0219	4.7445	0.0101	0.2952	0.3843	0.0069	0.0083	0.0045

16_1E	0.0133	8.1675	0.0064	0.2581	0.1976	0.0075	0.0043	0.0072
16_2E	0.0085	5.2531	0.0048	0.1283	0.0840	0.0032	0.0032	0.0019
16_3E	0.0133	5.7229	0.0123	0.2845	0.3683	0.0069		0.0059
18_1M	0.0123	0.6405	0.0064	0.0981	0.0197	0.0045	0.0043	0.0021
18_2M	0.0131	1.8816	0.0067	0.1323	0.0152		0.0043	0.0024
18_3M	0.0456	1.4125	0.0315	0.7331	0.0509	0.0093	0.0272	0.0123
18_1E	0.0421	1.9768	0.0248	0.6163	0.1208	0.0109	0.0179	0.0133
18_2E	0.0229	51.8211	0.0131	0.2973	0.0592	0.0075	0.0123	0.0053
18_3E	0.0461	53.8824	0.0259	0.7275	0.1096	0.0115	0.0147	0.0197
19_1M	0.0125	3.2859	0.0091	0.3648	0.3104	0.0085		0.0061
19_2M	0.0272	2.4501	0.0192	0.6504	0.5411	0.0168		0.0451
19_3M	0.0123	4.1363	0.0091	0.3379	0.2917	0.0093		0.0056
19_1E	0.0133	2.1835	0.0181	0.5776	0.5760	0.0168	0.0051	0.0240
19_2E	0.0189	1.4016	0.0056	0.5027	0.4989	0.0123	0.0064	0.0240
19_3E	0.0123	2.6461	0.0080	0.4957	0.5469	0.0128		0.0301

ID	Taurine	Threonine	Tyrosine	Urea	Uridine	Valine	sn-Glycero-3-phosphocholine	τ-Methylhistidine
4A_1M	0.1725	0.0523	0.0189	1.7741		0.0640	0.4096	0.0224
4A_2M	0.1731	0.0616	0.0248	1.8747	0.0131	0.0904	0.4475	0.0219
4A_3M	0.0656	0.0179		0.4248		0.0333	0.1349	
4A_1E	0.0203	0.0104		0.3083		0.0141	0.0568	
4A_2E	0.0685	0.0245		0.7741		0.0411	0.1813	
4A_3E	0.0128	0.0181	0.0133	0.4517		0.0315	0.0955	
4B_1M	0.1451	0.0357		1.6400		0.0264	0.4395	
4B_2M	0.0584	0.0405		1.2280	0.0101	0.0275	0.4587	
4B_3M	0.0504	0.0213		0.4005		0.0168	0.1699	
4B_1E		0.0019		0.3397		0.0069	0.0965	
4B_2E	0.0787	0.0205		0.9701		0.0139	0.3672	
4B_3E	0.0304	0.0067		0.2080		0.0040	0.0576	
5_1M	0.0469	0.0267		0.7565	0.0120	0.0227	0.2696	
5_2M	0.0627	0.0155		0.4856		0.0115	0.2784	
5_3M	0.0627	0.0256		0.6000		0.0152	0.2741	
5_1E	0.0419	0.0064		0.3760		0.0077	0.1464	
5_2E	0.1019	0.0275		1.4520		0.0192	0.4973	
5_3E	0.0456	0.0176		0.6784		0.0139	0.2659	
6A_1M				0.2749		0.0149	0.1307	
6A_2M	0.0571			1.0051		0.0371	0.3173	
6A_3M		0.0117		0.4592		0.0243	0.1832	
6A_1E	0.0453	0.0301		1.8552		0.0795	0.6035	0.0507
6A_2E	0.0501	0.0893	0.0397	2.2469		0.1112	0.6397	0.0541
6A_3E		0.0208		1.1419		0.0493	0.2163	0.0216
6B_1M	0.0512	0.0301		1.1400		0.0477	0.3723	0.0259
6B_2M	0.0787	0.0707	0.0251	1.4840		0.0629	0.5997	0.0291
6B_3M	0.1003	0.0632	0.0256	1.4043		0.0715	0.3485	0.0267
6B_1E	0.0208	0.0251		0.3501		0.0227	0.0981	
6B_2E		0.0395		1.4619		0.0643	0.3408	

6B_3E	0.0589	0.1056		2.3557		0.1051	0.4941	0.0645
6C_4M	0.0288	0.0176		0.4869		0.0107	0.1277	
6C_2M	0.0491	0.0685		2.0907		0.0411	0.4301	
6C_3M	0.0720	0.0181		0.5808		0.0120	0.1693	
6C_1E	0.0581	0.0323		1.2328		0.0192	0.2800	
6C_2E	0.1120	0.0253		0.4901		0.0149	0.1443	
6C_3E	0.1907	0.0480		2.1299		0.0269	0.5717	
6D_1M	0.1173	0.0755		0.8757		0.0333	0.2984	0.0160
6D_2M	0.0467	0.0477		0.9088		0.0288	0.2469	0.0187
6D_3M	0.1352	0.0944		1.7053		0.0499	0.4035	0.0243
6D_1E	0.0357	0.0395		0.6789		0.0189	0.1565	
6D_2E	0.0712	0.0560		1.0683		0.0336	0.2984	0.0248
6D_3E	0.0973	0.1000		2.4048		0.0451	0.4419	0.0267
7A_1M	0.0584	0.0309	0.0123	0.9328	0.0083	0.0253	0.2720	
7A_2M	0.0133	0.0125		0.2264		0.0085	0.0896	
7A_3M	0.0352	0.0064		0.1728		0.0059	0.0493	
7A_1E	0.0891	0.0568	0.0099	1.7571	0.0096	0.0352	0.3696	0.0259
7A_2E	0.0275	0.0240		0.4227		0.0141	0.1069	
7A_3E	0.0323	0.0189		0.4699		0.0139	0.0971	
7B_1M	0.0893	0.0571		0.7392		0.0293	0.4429	
7B_2M	0.0557	0.0568		0.7213		0.0293	0.4371	
7B_3M	0.0568	0.0763		1.3107		0.0429	0.7301	0.0267
7B_1E	0.0883	0.0379		0.4891		0.0168	0.3288	
7B_2E	0.0715	0.0603		0.9195		0.0288	0.3915	0.0133
7B_3E	0.0715	0.0920		1.1421		0.0397	0.7443	0.0293
8A_2M	0.0229	0.0099				0.0064	0.0501	
8A_3M	0.0645	0.0496		0.4789	0.0112	0.0173	0.2773	
8A_1E	0.0736	0.0515		0.6024	0.0131	0.0179	0.2523	
8A_3E	0.0379	0.0307	0.0088	0.4728	0.0123	0.0141	0.2573	
8B_1M	0.0296	0.0216		0.5107		0.0101	0.0997	
8B_2M	0.0493	0.0627	0.0171	1.7208		0.0437	0.4605	0.0272
8B_3M	0.0509	0.0171		0.5893		0.0109	0.1888	
8B_1E	0.1088	0.0515	0.0120	2.1069		0.0485	0.4987	0.0219
8B_2E	0.1285	0.0776	0.0173	2.7880		0.0549	0.4856	0.0312
8B_3E	0.0677	0.0771	0.0147	2.3560		0.0565	0.4827	0.0339
10_1M	0.0787	0.1456	0.0216	2.5152		0.0803	0.3613	0.0216
10_2M	0.0765	0.1424		2.2688		0.0757	0.3736	0.0221
10_3M	0.1197	0.1299	0.0139	2.3627		0.0725	0.3885	0.0267
10_1E	0.0539	0.0789		2.2701	0.0075	0.0512	0.3685	0.0216
10_2E	0.0501	0.0976	0.0197	2.2965		0.0533	0.4208	0.0187
10_3E	0.0597	0.0848		2.3893		0.0405	0.4067	0.0229
11_4M	0.2005	0.0941		2.0269	0.0163	0.0443	0.5605	
11_2M	0.0291	0.0208		0.6315		0.0128	0.0952	
11_3M	0.0539	0.0104		0.2901		0.0085	0.0691	
11_1E	0.0976	0.0629	0.0227	2.8136		0.0459	0.3005	0.0197
11_2E	0.0531	0.0379	0.0104	1.1792		0.0251	0.1744	

11_3E	0.0936	0.0245		1.6917		0.0157	0.2171	
13A_1M	0.0155	0.0440		0.4221		0.0133	0.1091	
13A_2M	0.0603	0.1213	0.0115	1.5256	0.0072	0.0312	0.4755	0.0235
13A_3M	0.0533	0.1029	0.0120	1.4917	0.0107	0.0296	0.4656	0.0211
13A_1E	0.0952	0.1304	0.0229	1.9008	0.0059	0.0371	0.3608	0.0373
13A_2E	0.0635	0.1013	0.0152	2.0072	0.0061	0.0325	0.3360	0.0235
13A_3E		0.1187	0.0128	1.5883	0.0088	0.0267	0.4603	0.0237
13B_1M	0.0461	0.0341		0.7160		0.0243	0.4195	
13B_2M	0.0371	0.0168		0.3469		0.0099	0.2040	
13B_3M	0.0629	0.0768		1.9947		0.0453	0.9688	0.0280
13B_1E	0.0757	0.1035		2.5048		0.0491	1.0920	0.0363
13B_2E	0.0915	0.1032		2.5989		0.0440	0.8789	0.0400
13B_3E	0.0640	0.0416		1.1816		0.0232	0.4613	
14_1M		0.0816		1.7205		0.0459	0.7003	
14_2M	0.1037	0.0365		1.1320	0.0083	0.0275	0.4896	
14_3M	0.0323	0.0109		0.2136		0.0053	0.1512	
14_1E	0.0528	0.0181		0.5056		0.0123	0.1923	
14_2E	0.1299	0.0419		2.1021	0.0088	0.0328	0.7837	
14_3E	0.1315	0.0592		2.0333	0.0107	0.0408	0.8595	
15A_1M	0.0539	0.1928	0.0336	1.4531	0.0459	0.0483	0.5781	0.0371
15A_2M	0.0437	0.0440		0.3997		0.0133	0.1125	
15A_3M	0.0981	0.1520		1.8525		0.0368	0.3789	0.0211
15A_1E	0.1077	0.1317	0.0144	1.5363		0.0304	0.3443	0.0219
15A_2E	0.1475	0.1059	0.0171	1.8011		0.0304	0.3291	0.0211
15A_3E	0.0392	0.0181		0.4581		0.0085	0.1008	
15B_1M	0.1581	0.0680	0.0189	2.3429	0.0101	0.0507	0.8955	0.0211
15B_2M	0.0731	0.0237		0.8173		0.0131	0.3235	
15B_3M	0.0592	0.0272		0.9944		0.0189	0.3971	
15B_1E	0.0696	0.0733	0.0189	3.1808		0.0501	1.4576	0.0669
15B_2E	0.0691	0.0459		1.9077		0.0320	0.6387	0.0221
15B_3E	0.0709	0.0432		2.1827		0.0269	0.7504	0.0133
15C_1M	0.1139	0.0443		1.8829	0.0083	0.0213	0.3867	
15C_2M	0.0840	0.0915	0.0429	3.3096	0.0253	0.0549	1.0968	
15C_3M	0.0211	0.0133		0.2357		0.0045	0.1115	
15C_1E	0.1560	2.6667		2.7952		0.0368	1.0712	
15C_2E	0.0555	0.0136		0.2539		0.0043	0.1611	
15C_3E	0.1949	0.0992		2.5400	0.0197	0.0456	0.7987	0.0467
16_1M	0.0299	0.0027		0.2888			0.1032	
16_2M	0.0523	0.0360		2.1029		0.0141	0.8019	
16_3M	0.0613	0.0323		0.7517		0.0149	0.3645	
16_1E	0.0523	0.0219		0.8541		0.0123	0.3355	
16_2E	0.0413	0.0056		0.3800		0.0048	0.1547	
16_3E	0.0523	0.0261		0.7075		0.0173	0.3421	
18_1M	0.0256	0.0048		0.2656		0.0051	0.0125	
18_2M	0.0344	0.0064		0.2469			0.0109	
18_3M	0.1448	0.0253	0.0125	1.5837		0.0232	0.0115	

18_1E	0.0611	0.0336	0.0099	1.7331		0.0267	0.1416
18_2E	0.0411	0.0251		0.7640		0.0141	0.0944
18_3E	0.1440	0.0395	0.0160	2.1677		0.0307	0.2419
19_1M	0.0595	0.0293		0.9040		0.0195	0.4128
19_2M	0.0917	0.0627	0.0152	1.7197		0.0283	0.6205
19_3M	0.0523	0.0221		0.8939		0.0120	0.3403
19_1E	0.0568	0.0376		2.5253		0.0243	0.7771
19_2E	0.0659	0.0325	0.0112	2.0211		0.0237	0.6115
19_3E	0.0824	0.0384	0.0120	1.9368		0.0275	0.7160

Table S4. Polar metabolite concentration in the formula milk samples (blank boxes mean that relevant metabolite was not detected in the corresponding sample).

ID	2-Oxoglutarate	3-SL	Acetate	Acetone	Alanine	Betaine	Butyrate	Carnitine	Choline
A 1		0.0083	0.0288	0.0157	0.0131	0.0789		0.0515	0.5445
A 2	0.0392	0.0037	0.0240	0.0088	0.0107	0.0797	0.0109	0.0344	0.6789
A 3	0.0235	0.0048	0.0349	0.0088	0.0099	0.0331		0.0141	0.1275
B 1		0.0032	0.0125	0.0067	0.0056	0.0291		0.0264	0.2733
B 2	0.0307	0.0035	0.0344	0.0051	0.0096	0.0229		0.0216	0.3621
B 3		0.0040	0.0179	0.0096	0.0059	0.0203		0.0301	0.0637
C 1	0.0163	0.0032	0.0315	0.0040	0.0053	0.0224		0.0299	0.4195
C 2	0.0064		0.0203	0.0045	0.0048	0.0131		0.0093	0.2963
C 3	0.0139		0.0115	0.0045	0.0051	0.0136		0.0056	0.1568
D 1	0.0133	0.0077	0.0864	0.0048	0.0040	0.0603		0.0216	0.5592
D 2	0.0189	0.0067	0.0664	0.0099	0.0155	0.0461		0.0360	0.0600
D 3	0.0104		0.0173	0.0043	0.0053			0.0061	0.0227
D 1	0.0352		0.0213	0.0021	0.0205			0.0184	0.2304
D 2	0.0408		0.0467	0.0045	0.0395	0.0309		0.0251	0.0443
D 3			0.0445	0.0016	0.0307			0.0269	2.6667
E 1	0.0219		0.0088	0.0019	0.0136	0.0317		0.0117	0.4648
E 2	0.0472	0.0032	0.0147	0.0053	0.0229	0.0736		0.0456	1.0733
F 1	0.0240		0.0123	0.0016	0.0133			0.0101	0.2472
F2	0.0107		0.0125	0.0011	0.0120			0.0096	0.0224

ID	Citrate	Creatine	Creatine phosphate	Creatinine	Dimethylamine	Formate	Fumarate	Galactose	Gluconate
A 1	2.8320	0.3173		0.0736	0.0061	0.0381	0.0053	2.9957	0.0725
A 2	2.6696	0.2152		0.0539	0.0051	0.0435	0.0069	0.9032	0.0547
A 3	4.2208	0.1875		0.0563	0.0045	0.0568	0.0051	2.1317	0.0424
B 1	1.7872	0.1523		0.0419	0.0032	0.0259	0.0021	2.3712	0.0739
B 2	1.9768	0.1288		0.0325	0.0040	0.0363	0.0064	0.4931	0.0587

B 3	3.2813	0.1616		0.0453	0.0027	0.0285	0.0035	0.9157	0.0424
C 1	1.2896	0.0861		0.0256	0.0032	0.0275	0.0056	0.1499	0.0459
C 2	0.8923	0.0795		0.0192	0.0024	0.0253	0.0027	0.1829	
C 3	1.0464	0.0597		0.0131	0.0029	0.0195	0.0024	0.1157	
D 1	2.5069	0.0765		0.0099	0.0035	0.0395	0.0035	1.5688	0.0173
D 2	4.0053	0.1181		0.0195	0.0048	0.0645	0.0045	0.5731	0.0499
D 3	1.1317	0.0440		0.0077	0.0021	0.0211	0.0027	0.2389	
D 1	1.7779	0.2509		0.0331	0.0045	0.0533	0.0051	0.3360	0.0259
D 2	3.0451	0.3672		0.0712	0.0069	0.0840	0.0067	0.5720	0.0512
D 3	2.3717	0.3368		0.0451	0.0045	0.0571	0.0067	0.4251	
E 1	1.2357	0.1136	0.0144	0.0171	0.0024	0.0253	0.0024	0.3304	0.0197
E 2	2.5803	0.2501	0.0371	0.0512	0.0061	0.0499	0.0059	0.8864	0.0509
F 1	1.2032	0.1752		0.0331	0.0024	0.0379	0.0029	0.2080	0.0365
F2	1.0776	0.1157		0.0208	0.0011	0.0317	0.0027	0.1827	2.6667

ID	Glucose	Glutamate	Hippurate	Isoleucine	Lactate	Lactose	Malonate	Maltose	Methanol
A 1	12.6816	0.1347	0.0248		0.1888	155.4059	0.0581		515.0675
A 2	11.3040	0.0760	0.0773	0.0853	0.0507	168.3285	0.0419		234.8373
A 3	13.3101	0.0744	0.0525		0.1472	125.1784		6.9915	67.1885
B 1	7.1637	0.0360	0.0160		0.1152	80.8192			172.0472
B 2	7.3165	0.0613	0.0224		0.1776	99.8197		7.5867	38.5531
B 3	8.7216		0.0285		0.1509	74.9557		6.1445	359.1547
C 1	1.7224	0.0472	0.0200		0.2901	68.6352	0.0096		43.6781
C 2	2.8501	0.0381	0.0400		0.0608	27.1621	0.0083	9.5763	163.3197
C 3	1.7499	0.0413	0.0149		0.0485	18.6536	0.0075	8.4229	0.8891
D 1		2.6667			1.2640	122.0744			69.8765
D 2	0.4344	0.0557	0.0245		0.2040	120.9381	0.0109		13.1528
D 3	0.3317	2.6667			0.0715	23.0128		3.5221	19.9125
D	1.1584	0.0632	0.0371	0.3043	0.2003	72.7328	0.0131	12.4181	6.7381

1									
D 2	2.1368	0.0813	0.0800	0.2805	0.3293	110.2365	0.0128	21.8813	84.7275
D 3	1.4875		0.0576	0.3392	0.4240	94.1800		13.1283	578.9491
E 1		0.0299	0.0381	0.1411	0.0432	76.9549		1.3067	48.0451
E 2		0.0704	0.0632	0.3072	0.0651	157.5920	0.0149		74.2416
F 1	0.8965	0.0213	0.0283	0.1883	0.1269	48.2083		7.2805	62.2155
F2	0.6416		0.0184	0.0733	0.1160	36.5056		7.0005	161.8680
**ID**	**Methionine**	**N-Acetylglucosamine**	**O-Phosphocholine**	**Pantothenate**	**Phenylalanine**	**Pyruvate**	**Succinate**	**Sucrose**	**Taurine**
A 1	0.0307	0.1027	0.0989	0.0272		0.0117	0.0059		
A 2	0.0197	0.0528	0.0293	0.0152		0.0075	0.0131		
A 3	0.0173	0.0573	0.0488	0.0203		0.0083	0.0112	0.6643	
B 1	0.0189	0.0584	0.0555	0.0120		0.0045	0.0040		
B 2	0.0093	0.0600	0.0384	0.0152		0.0088	0.0064		
B 3	0.0179	0.0432	0.0699	0.0093		0.0059	0.0061		
C 1	0.0072	0.0627	0.0245	0.0112		0.0045	0.0096		
C 2	0.0061	0.0179	0.0213	0.0072		0.0032	0.0107		
C 3	0.0043	0.0163	0.0176	0.0048		0.0035	0.0032	4.9613	
D 1	0.0115	0.0797	0.0656	0.0251	0.7464	0.0115	0.0040		0.0560
D 2	0.0216	0.0680	0.0464	0.0221		0.0091	0.0083		
D 3	0.0059	0.0160	0.0131	0.0067		0.0037	0.0021		
D 1	0.5680	0.0248	0.1323	0.0227		0.0203	0.0203		
D 2	0.0195	0.0317	0.2080	0.0261		0.0272	0.0379		
D 3	0.0133	0.0189	0.1755	0.0259		0.0147	0.0445		
E 1	0.0067	0.0171	0.0715	0.0112		0.0069	0.0171		0.1795
E 2	0.0211	0.0512	0.1797	0.0195		0.0133	0.0339		0.4128
F 1	0.3464	0.0155	0.0909	0.0189		0.0104	0.0187		
F2	0.0064	0.0115	0.0675	0.0120		0.0112	0.0117		
**ID**	**Tryptophan**	**UDP-galactose**	**UDP-glucose**	**Urea**	**Uridine**	**Valine**	**sn-Glycero-3-phosphocholine**		

**A 1**	0.0195			0.4072	0.0155		0.6112
**A 2**	0.1389			0.6709	0.0099		0.2720
**A 3**				0.2899			0.3491
**B 1**							0.3371
**B 2**	0.0299				0.0085		0.1787
**B 3**				0.1549	0.0107		0.2275
**C 1**							
**C 2**				0.1152			0.1125
**C 3**				0.1405			0.1005
**D 1**					0.0141		0.5984
**D 2**				0.3456			0.3795
**D 3**							0.1107
**D 1**	0.8035	0.1181	0.0507	0.4608	0.0709	0.0307	0.1171
**D 2**	0.5056	0.1600	0.1067	0.7837	0.1037	0.0381	0.1976
**D 3**	0.4597	0.1581	0.0928	0.7075	0.0688	0.0275	0.1499
**E 1**	0.1579	0.0728	0.0381	0.3192	0.0157	0.0093	0.0608
**E 2**	0.4395	0.1589	0.0840	0.7453	0.0365	0.0141	0.1160
**F 1**	0.5056	0.0795	0.0419	0.3640	0.0267	0.0133	0.0707
**F2**	0.1565	0.0547	0.0296	0.2075	0.0291	0.0107	0.0563
